# Ultrasound‑enhanced green recovery and antioxidant potential of polyphenols from four *Abelmoschus manihot* bio‑wastes

**DOI:** 10.1016/j.ultsonch.2026.108145

**Published:** 2026-11-04

**Authors:** Wen-zhang Qian, Li Zhao, Jin-you Yao, Pei-wen Liang, Yu-han Hu, Yi-xiao Xiao, Shun Gao

**Affiliations:** a Department of Forestry, Faculty of Forestry, Sichuan Agricultural University, Chengdu 611130, China; b Forest Ecology and Conservation in the Upper Reaches of the Yangtze River Key Laboratory of Sichuan Province & Sichuan Mt. Emei Forest Ecosystem National Observation and Research Station, Sichuan Agricultural University, Chengdu 611130, China

**Keywords:** *Abelmoschus manihot*, Ultrasonic-assisted extraction, Green recovery, Polyphenol, UHPLC‑MS/MS metabolomics, Antioxidant activity

## Abstract

*Abelmoschus manihot* (*A. manihot*) flowers are well studied, but its fruit, leaf, stem, and root bio-wastes remain underutilized. This study developed a green ultrasonic-assisted extraction (UAE) process using a temperature-controlled bath (40 kHz, 12 L). Calorimetric calibration established linear acoustic energy output (R^2^ = 0.9942), and a medium acoustic energy density of 113.6 W/L was selected. This energy density ensured stable cavitation during extraction. KI dosimetry quantified cavitation: triiodide concentration increased with power above a threshold, and time-course data were fitted by both linear and nonlinear models, confirming continuous sonochemical activity. Water, ethanol, and water/ethanol (1:1, v/v) were compared under UAE and shake-assisted extraction. Water/ethanol with UAE gave the highest polyphenol yields and antioxidant activities, with a net increment of 3.87 mg GAE g^−1^ for leaf. Pseudo-second-order kinetics best described extraction, and UAE increased rate constants, indicating reduced mass transfer resistance. SEM revealed ultrasound-induced cell wall disruption, more pronounced in leaf and fruit than in lignified stem and root. Extraction parameters were optimized by response surface methodology and back-propagation artificial neural network, with the latter superior. Antioxidant assays ranked fruit > leaf > stem > root. UHPLC-MS/MS metabolomics identified 508 polyphenols dominated by flavonoids (57.75 %) and phenolic acids (38.76 %), with tissue-specific accumulation. Mantel test showed flavonoids and phenolic acids strongly correlated with antioxidant activity, with tannins playing auxiliary roles. This integrated acoustic, kinetic, and microstructural evidence provides a mechanistic basis for ultrasound-enhanced polyphenol recovery. These findings demonstrate the greenness and potential of UAE for recovering polyphenols from agricultural bio-wastes.

## Graphical abstract

**Figure:**
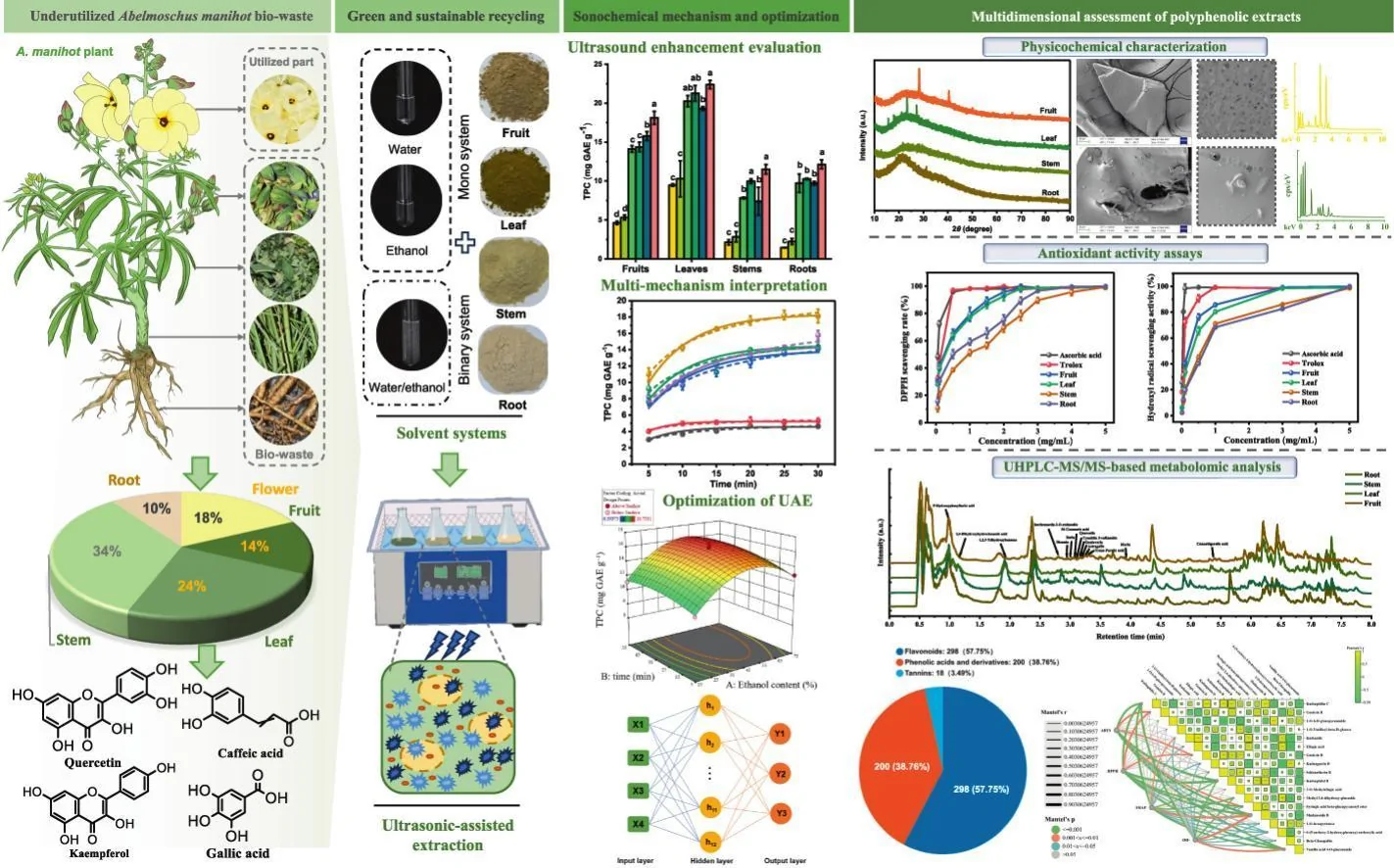

## 1 Introduction

*Abelmoschus manihot* L. Medicus (*A. manihot*) is a perennial plant with edible, medicinal and economic value, widely distributed in Asia, Eastern Europe and South Pacific island countries [1], [2]. Its flowers have been used as a traditional Chinese medicine for hundreds of years, with effects such as clearing heat, detoxifying, and removing dampness [3], [4], [5]. Modern pharmacological studies have confirmed that the whole plant is rich in flavonoids, phenolic acids, polysaccharides, steroids and other natural active components [6], [7], [8]. At present, most research and commercial development focus on the flowers (Supplementary Fig. 1**A**) [6], [9]. However, the roots, stems, leaves and fruits, which account for more than 80 % of the plant biomass, have long been not effectively utilized (Supplementary Fig. 1**B**) [8], [10]. They are often discarded or burned directly [10]. This crude treatment causes great waste of high‑value functional biomass and leads to environmental problems such as solid waste accumulation and air pollution [8], [10], [11]. With the global concept of green development and the recycling of agricultural resources, the green transformation and high‑value utilization of such bio‑waste have become research hotspots in the fields of natural products and agricultural processing [12], [13], [14], [15]. Therefore, studying the green extraction and functional evaluation of active substances from *A. manihot* processing bio-waste is important for the whole‑plant utilization, industrial chain extension and added‑value improvement.

Polyphenols are plant secondary metabolites with multiple biological activities such as antioxidant, anti‑inflammatory, antibacterial and cardiovascular protection [4], [16], [17], [18]. They have broad application prospects in food, medicine and cosmetics [19], [20]. *A. manihot* is rich in polyphenols including quercetin, kaempferol, caffeic acid and gallic acid. These components are considered the key material basis for its antioxidant and health‑promoting effects (Supplementary Fig. 1**C**) [3], [4], [7]. Polyphenols are heat‑sensitive and easily degraded, so the choice of extraction method directly determines the extraction yield, activity retention and environmental friendliness of the process [16], [21], [22]. Traditional extraction methods such as cold soaking and heat reflux extraction usually have disadvantages including high solvent consumption, long extraction time and easy thermal degradation of active components [21], [23], [24], [25]. Traditional organic extraction solvents (such as methanol, acetone and ethyl acetate) possess good solubility, but suffer from toxic residues, poor environmental compatibility and high cost [23], [24], [25]. Neither these conventional extraction approaches nor toxic organic solvents satisfy the basic principles of green chemistry. In contrast, UAE is a typical green intensified technology [26], [27], [28]. It relies on the mechanical shock waves and micro‑jets generated by ultrasonic cavitation to disrupt plant cell wall structures, thereby improving solvent penetration and mass transfer of target compounds [29], [30], [31]. UAE has advantages such as fast extraction, low energy consumption, low solvent usage and high retention of active substances [28], [32], [33], [34], [35]. It has been widely used to extract polyphenols and flavonoids from various plants [28], [36]. The efficiency of UAE is governed by the interplay of multiple factors, including ultrasonic parameters, solvent properties, and plant matrix characteristics [37]. Proper selection of a green solvent system is also a key step for efficient and sustainable polyphenol extraction [15], [38], [39], [40]. In recent years, the green-solvent landscape has become increasingly nuanced. Deep eutectic solvents (DES) combined with UAE have emerged as highly efficient platforms for extracting bioactive polysaccharides and flavonoids from medicinal plant wastes, owing to their highly tunable properties and low volatility [35], [36], [37]. However, despite their high extraction efficiency, the inherent high viscosity of DES often restricts mass transfer during extraction and complicates downstream product separation, making solvent recovery highly energy-intensive [12], [41], [42]. Among green alternatives, water and ethanol stand out not merely as a conventional choice, but as a rationally optimized extraction medium. They offer outstanding advantages such as safety, low toxicity, low cost, easy availability, and low viscosity for facile mass transfer [39], [41]. A water/ethanol binary system offers complementary advantages: water promotes cell wall swelling through hydrogen-bond disruption, while ethanol facilitates dissolution of medium-polarity polyphenols [33], [34]. In contrast, pure water has limited ability to solubilize less polar phenolic compounds, and anhydrous ethanol lacks sufficient swelling capacity to effectively penetrate lignocellulosic networks [7], [35], [36]. In addition to solvent effects, plant tissues differ considerably in their structural resistance to ultrasonic disruption. Tissues with thin, non-lignified cell walls are generally more susceptible to cavitation-induced damage, whereas lignin- and cellulose-rich tissues exhibit greater rigidity, which may limit extraction efficiency under identical ultrasonic conditions [7], [37]. Kinetic modeling provides a quantitative means to characterize ultrasound-induced mass transfer enhancement, with the pseudo-second-order model and pseudo-first-order model often offering a good description of solid–liquid extraction processes [7], [33], [37]. Microscopic evidence has shown that cavitation can generate surface erosion, pore formation, and cell wall rupture, all of which are associated with improved release of intracellular compounds [7], [32]. These mechanistic considerations suggest that the synergy between ultrasound, solvent polarity, and matrix structure is a key determinant of extraction efficiency. Therefore, a systematic investigation of these interacting factors is essential for the rational design and optimization of UAE processes in natural product recovery.

However, combining UAE with green solvents alone is insufficient to fully exploit the resource potential of *A. manihot* bio-waste. Most extraction studies have focused on a single tissue, such as flowers or leaves [6], [8], [10], and systematic reports on process optimization and graded extraction across different waste organs remain scarce. Given that cell wall structure, lignification degree, and secondary metabolite accumulation vary significantly among tissues [39], [40], [43], a “one-size-fits-all” extraction strategy would inevitably lead to incomplete extraction or loss of active components in certain tissues. From a whole-industry-chain perspective, tailoring extraction conditions to each waste organ can enable graded utilization based on tissue-specific polyphenol profiles, thereby increasing the overall added value of the whole plant resource [12], [14], [39], [44]. More importantly, differences in secondary metabolic regulation result in significant variation in polyphenol composition, types, and contents among organs [39], [44]. Using only *in vitro* antioxidant assays can quickly evaluate the functional potential of the extracts, but it is difficult to accurately identify the specific chemical identities and contribution weights of the active substances [20], [45]. Therefore, it is urgent to use UHPLC‑MS/MS metabolomics to perform comprehensive and unbiased qualitative and quantitative analysis of complex polyphenol systems, and to systematically characterize their chemical composition and relative abundance [46], [47]. By precisely characterizing the component profiles and characteristic chemical components of extracts from different parts, we can establish the internal relationship among source, composition and activity, thus providing a chemical basis for the targeted and refined utilization of different wastes.

Although our previous research has explored the comprehensive utilization of *A. manihot* flowers [6], [7], [9], and Yang et al. [8] and Zhao et al. [10] optimized the extraction of flavonoids from *A. manihot* leaves using ultrasonic‑enzyme‑assisted deep eutectic solvent extraction and enzyme pretreatment combined with ultrasound‑assisted aqueous two‑phase extraction, respectively, systematic studies on green polyphenol extraction, multi‑model synergistic optimization, metabolomic changes and polyphenol‑antioxidant structure‑activity relationships for all *A. manihot* bio-wastes (leaves, fruits, stems and roots) are still lacking. Therefore, this study uses four types of agricultural bio-wastes of *A. manihot* as raw materials and employs a green UAE technology to enrich polyphenols. Solvent systems and extraction modes are systematically compared to identify the optimal extraction conditions. Kinetic modeling is applied to quantitatively evaluate the mass transfer behavior under ultrasonic treatment. Scanning electron microscopy is used to observe microstructural changes of the plant matrix before and after ultrasound treatment. Single-factor experiments combined with mathematical modeling approaches are used for multi-parameter optimization to determine the extraction conditions for each tissue. UHPLC‑MS/MS metabolomics is used to analyze the differences in polyphenol composition among different parts. Four *in vitro* antioxidant assays (DPPH, ABTS, FRAP and hydroxyl radical scavenging) are used to systematically evaluate the bioactivity of the extracts. The complete technical route is shown in Supplementary Fig. 1**D**. This study aims to establish a green ultrasound‑based polyphenol extraction system for *A. manihot* bio-wastes, and to clarify the organ‑specific distribution and antioxidant potential of endogenous polyphenols. The results can provide a theoretical basis and practical solutions for the high‑value utilization of *A. manihot* bio-wastes, and also serve as a reference for the green development and multi‑model optimization of extraction processes for other medicinal and edible plant wastes.

## 2 Materials and methods

### 2.1 Samples and reagents

*A. manihot* plant material used in this study was collected from Jingshan Village, Yong'an Town, Shuangliu District, Chengdu, Sichuan Province, China (latitude 30°36′59.54″ N, longitude 104°00′41.14″ E). The post‑flowering waste plants were collected in the field and transported to the laboratory. As illustrated in Supplementary Fig. 1**A**, the waste plants were separated into four parts: fruit, leaf, stem, and root. Each part was dried to constant weight in a forced‑air oven at 65 °C, then crushed, ground into a homogeneous powder, and passed through a 60‑mesh sieve for subsequent use. All chemical reagents used in the experiments were of analytical grade unless otherwise specified. Anhydrous ethanol, methanol, hydrochloric acid, sodium hydroxide, and sodium chloride were purchased from Sinopharm Chemical Reagent Co., Ltd. (China). ABTS, DPPH, potassium persulfate, and Trolox were obtained from Sigma‑Aldrich (USA). Folin–Ciocalteu reagent, gallic acid, ferrous sulfate heptahydrate, ferric chloride hexahydrate, sodium acetate, acetic acid, sodium dihydrogen phosphate, and disodium hydrogen phosphate were purchased from Thermo Fisher Scientific (USA). 2,4,6‑Tri(2‑pyridyl)‑1,3,5‑triazine (TPTZ), and the hydroxyl radical detection reagents were of analytical grade. For UHPLC‑MS/MS metabolomics analysis, chromatographic‑grade methanol and acetonitrile were purchased from Merck (Germany), and chromatographic‑grade formic acid was obtained from Aladdin Biochemical Technology Co., Ltd. (China).

### 2.2 Ultrasonic extraction system and experimental design

UAE experiments were performed using a temperature-controlled digital ultrasonic cleaning bath (Model JP-080ST, Shenzhen Jiemeng Technology Co., Ltd., China) with a fixed operating frequency of 40 kHz, was selected according to our prior study, in which efficient polyphenol extraction from *A. manihot* bio-wastes was obtained. The internal dimensions of the ultrasonic tank were 60 cm × 40 cm, with an effective liquid filling volume of 12 L. To eliminate thermal artifacts from ultrasonic irradiation, the bath was coupled with a constant-temperature recirculating chiller (5–30 °C), and three temperature probes were deployed to monitor the ultrasonic bath medium and sample liquid phase, respectively (Fig. 1**A**). Real-time temperature monitoring was implemented throughout sonication (Fig. 1**B**). Calorimetry was used to calibrate the linear relationship between the instrument's set output power and the actual acoustic energy density absorbed by the liquid medium (Fig. 1**C**), following the temperature rise method [39] using the equation (1):

(1) $$P=\frac{\left(m{\times C}_{p}\times \Delta T\right)}{t}$$

**Fig. 1:**
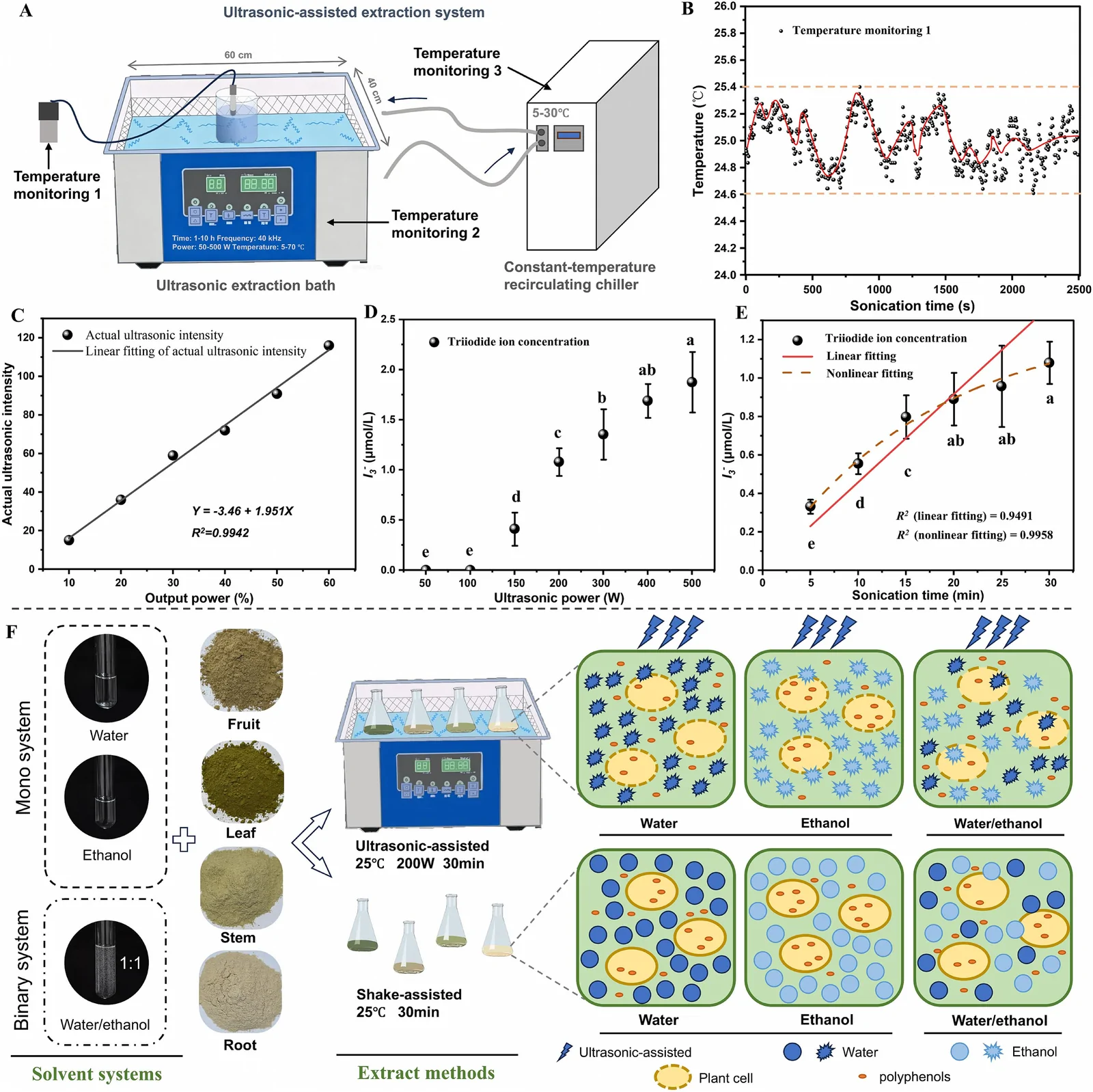
Schematic, energy calibration, cavitation quantification and experimental design of the UAE system for polyphenol extraction from *A. manihot*. (A) Schematic of temperature-controlled UAE system fitted with circulating chiller and triple temperature probes. (B) Real-time temperature monitoring during ultrasonication. (C) Linear correlation between actual ultrasonic intensity and equipment output power of the ultrasonic bath. (D) Triiodide (*I_3_^−^*) concentration obtained *via* KI dosimetry at different ultrasonic power (30 min sonication), representing cavitation activity. (E) Time-course accumulation of triiodide measured by KI dosimetry with linear and nonlinear fitting. (F) Experimental setup of mono/binary solvents, four *A. manihot* tissues, and mechanistic schematic comparing polyphenol release under UAE and shake-assisted extraction across three solvent media.

where *m* is the water mass (g), *C_p_* is the specific heat capacity (4.18 J g^−1^ K^−1^), *ΔT* is the temperature increase (K), and *t* is the irradiation time (s).

Potassium iodide (KI) dosimetry was performed following the procedure described by Mason et al. [38] to quantify the ultrasonic cavitation activity of the extraction system, using the same temperature-controlled ultrasonic bath and reaction vessel as in the extraction experiments, with a liquid filling volume of 100 mL and the bath temperature maintained at 25 ± 1 °C by an external recirculating chiller. A freshly prepared 0.1 mol/L KI aqueous solution (16.6 g KI in 1 L ultrapure water, stored in a brown bottle protected from light) was sonicated under two sets of conditions: (i) fixed sonication time of 30 min with varying ultrasonic power (100, 200, 300, 400, and 500 W), and (ii) fixed ultrasonic power of 200 W with varying sonication time (5, 10, 15, 20, 25, and 30 min). After sonication, the absorbance of the solution was immediately measured at 352 nm against an unsonicated blank using a Shimadzu UV-1900 spectrophotometer, and the triiodide (*I_3_^−^*) concentration was calculated according to the Beer–Lambert law using equation (2):

(2) $$c\left(I_{3}^{-}\right)=\frac{A}{\varepsilon ∙l}$$

where *A* is the absorbance at 352 nm, *ε* is the molar absorption coefficient of *I_3_^−^* (26,000 L mol^−1^ cm^−1^), and *l* is the optical path length (1 cm). All measurements were conducted in triplicate, and the time-course triiodide accumulation data were subsequently fitted with linear and nonlinear models to characterize the sonochemical activity.

A full-factorial experimental design was established to systematically compare three solvent systems (water MS, ethanol MS, and water/ethanol BS, 1:1 v/v) and two extraction modes, stirring-assisted extraction (NUAE) and UAE, for polyphenol recovery from four *A. manihot* bio-wastes (fruit, leaf, stem, root). For each batch, 1.0 g of dried powder was mixed with solvent at a fixed liquid–solid ratio (LSR) of 30:1 (mL/g) and thermostabilized at 25 °C for 30 min. NUAE groups were agitated at 200 rpm without ultrasonic exposure, while UAE groups were treated at 40 kHz and 200 W in the calibrated bath, with the chiller maintaining constant temperature. After extraction, suspensions were centrifuged at 10,000 rpm for 5 min at 4 °C, and supernatants were analyzed for TPC and antioxidant activities. The overall experimental workflow and schematic illustration of extraction processes in different solvent systems under NUAE and UAE are shown in Fig. 1**F**.

### 2.3 Extraction kinetics and model fitting

Time-series extraction experiments were conducted for all four tissues under six solvent-extraction combinations (Supplementary Table 1.). Samples were collected at 5, 10, 15, 20, 25, and 30 min under calibrated conditions (40 kHz, 200 W, 25 °C, LSR = 30:1 mL/g), centrifuged, and analyzed for TPC to construct extraction curves. Pseudo-first-order and pseudo-second-order kinetic models were fitted to the time-series TPC data for each group using both nonlinear regression and linear regression approaches. The pseudo-first-order model assumes that the extraction rate is proportional to the distance from equilibrium, expressed as equation (3):

(3) $$Y_{t}=Y_{e}-Y_{e}e^{-k_{1}t}$$

Linear transformation of the pseudo-first-order model was performed as equation (4):

(4) $${ln(Y_{e}-Y}_{t})={ln(Y}_{e})-k_{1}t$$

where *Y_t_* (mg GAE g^−1^) is the TPC at time t (min), *Ye* (mg GAE g^−1^) is the theoretical equilibrium TPC, and *k_1_* (min^−1^) is the pseudo-first-order rate constant.

The pseudo-second-order model assumes that the extraction rate is controlled by the availability of accessible solute-binding sites on the solid matrix and is given by equation (5):

(5) $$\frac{t}{Y_{t}}=\frac{1}{k_{2}Y_{e}^{2}}+\frac{t}{Y_{e}}$$

Where *k_2_* (g mg^−1^ min^−1^) is the pseudo-second-order rate constant. The rate constants (*k_1_*, *k_2_*) and theoretical equilibrium TPC (*Y_TPC_*) derived from model fitting served as quantitative indicators of ultrasound-induced mass transfer enhancement, avoiding unsubstantiated speculative descriptions of unmeasured cavitation phenomena. All kinetic parameters were tabulated for subsequent comparison across tissues and solvent systems. To rigorously evaluate the goodness-of-fit and statistical robustness, non-linear least-squares regression was applied, and all kinetic parameters along with their 95 % confidence intervals and Root Mean Square Errors (RMSE) were comprehensively tabulated (Supplementary Table 2) for subsequent comparison across tissues and solvent systems.

The triiodide concentration and its formation rate obtained from KI dosimetry experiments under the UA-W MS system were adopted for correlation analysis with TPC extraction data of four *A. manihot* bio-wastes. Linear regression was performed to fit the relationship between equilibrium TPC yield (*Y_TPC_*) and steady-state triiodide concentration ([*I_3_^−^*]), as well as the correlation between *dY_TPC_/dt* and d[*I_3_^−^*]/d*t*. Pearson correlation coefficients between *dY_TPC_/dt* and d[*I_3_^−^*]/d*t* were calculated.

### 2.4 Experimental design for UAE of polyphenols from bio-wastes of *a. Manihot*

#### 2.4.1 Single‑factor experiments

To investigate the effects of four key process parameters, ethanol concentration, ultrasound irradiation time, LSR, and ultrasound power, on the extraction of polyphenols from bio-wastes of *A. manihot* and their antioxidant activities, single‑factor experiments were conducted. The experiments were based on UAE, with the baseline conditions set as follows: ethanol content 95 % (v/v), ultrasound time 30 min, LSR 30:1 mL/g, ultrasound power 200 W, and a constant extraction temperature of 37 °C. When examining the influence of a single factor, all other parameters were kept at the above baseline levels. The tested levels for each factor were: ethanol concentration (15, 30, 45, 60, 75, and 95 % v/v), ultrasound time (0, 5, 15, 30, 45, and 60 min), LSR (15:1, 30:1, 45:1, 60:1, 75:1, and 90:1 mL/g), and ultrasound power (50, 100, 200, 300, 400, and 500 W).

#### 2.4.2 Determination of optimal extraction conditions

Based on the results of the single‑factor experiments, three key parameters, ethanol content (A), ultrasound time (B), and LSR (C) were selected as independent variables for optimization. The response values were TPC, DPPH radical scavenging activity, and ABTS radical scavenging activity. A three‑factor, three‑level Box–Behnken design was employed to construct a response surface methodology model for optimizing the UAE of polyphenols from different bio-wastes of *A. manihot*. The coded and actual levels of the variables are listed in Supplementary Table 3, and the complete design matrix consisting of 17 experimental runs is presented in Supplementary Table 4. Multiple regression analysis was performed using Design‑Expert 13 software to fit the experimental data to a second‑order polynomial model, which included linear, quadratic, and interaction terms of the independent variables. Analysis of variance (ANOVA) was conducted to evaluate the statistical significance of the model and individual terms, including main effects, interaction effects, and quadratic effects. The adequacy of the model was further assessed using the coefficient of determination (R^2^), adjusted R^2^, and predicted R^2^.

A three-layer back-propagation artificial neural network (BP-ANN) was constructed as a complementary data-driven tool to model the relationship between extraction parameters and responses. The input layer comprised four variables (ethanol content, ultrasound time, LSR, and ultrasound power), and the output layer comprised three responses (TPC, DPPH, and ABTS). A total of 120 datasets per tissue, derived from single-factor and Box-Behnken design experiments, were used. To ensure reproducibility, a fixed random seed was applied, and the data were randomly divided into training (70 %), validation (15 %), and test (15 %) sets. All data were normalized to [1] prior to training. The hidden layer neuron number was optimized by trial-and-error within 4–20 neurons based on validation error. The sigmoid and linear transfer functions were used for the hidden and output layers, respectively. The network was trained using the back-propagation algorithm with mean squared error as the loss function. To mitigate the risk of overfitting, an early stopping criterion was implemented, where training was halted if the validation error continuously increased for six consecutive epochs. Model performance was evaluated using R^2^, MSE, RMSE, MAE, and MAPE for all data subsets.

### 2.5 Determination of TPC

TPC was determined using the Folin–Ciocalteu method with gallic acid as the standard, based on our previously reported method [6]. Gallic acid standard solutions (0–120 mg/L) were prepared in ultrapure water. Briefly, 1.0 mL of standard or sample was mixed with 5.0 mL Folin–Ciocalteu reagent, vortexed, and left for 3 min at room temperature. Then, 4.0 mL of 7.5 % Na_2_CO_3_ was added, vortexed, and the mixture was incubated in the dark for 60 min. Absorbance was read at 765 nm against a blank (ultrapure water). TPC was calculated from a calibration curve and expressed as mg gallic acid equivalents per gram dry weight (mg GAE·g^−1^ DW).

### 2.6 *In vitro* antioxidant activity assays

Extracts from different bio-wastes of *A. manihot* were reconstituted in ultrapure water or absolute ethanol to prepare a series of concentration gradients, based on our previously reported method [7]. The solutions were centrifuged at 8000g for 10 min, and the supernatants were filtered through a 0.22 μm organic membrane. The filtrates were used for antioxidant activity assays. Ascorbic acid (Vc) and Trolox were used as positive controls.

#### 2.6.1 DPPH radical scavenging activity

The DPPH radical scavenging activity was determined according to our previous method with slight modifications [6]. A 0.1 mmol/L DPPH ethanol solution was prepared and stored in the dark. Then, 1.5 mL of sample solution at various concentrations was mixed with an equal volume of DPPH solution, vortexed, and incubated in the dark at room temperature for 30 min. The absorbance was measured at 517 nm (as using ultrapure water as a blank. Two control sets were prepared: (i) 1.5 mL DPPH solution + 1.5 mL absolute ethanol (absorbance A_1_), and (ii) 1.5 mL sample solution + 1.5 mL ultrapure water (absorbance A_2_). The DPPH radical scavenging activity was calculated using equation (6):

DPPH radical-scavenging activity (%) = [1-(A_1_- A_2_)/A_3_] *100 (6).

A concentration–scavenging rate curve was plotted, and the half‑maximal inhibitory concentration (IC_50_) value indicates stronger DPPH radical scavenging capacity.

#### 2.6.2 ABTS radical scavenging activity

Based on our previously reported method [7], the ABTS working solution was prepared as follows: 7 mmol/L ABTS solution and 2.45 mmol/L potassium persulfate solution were mixed at 1:1 (v/v) and kept in the dark at room temperature for 12–16 h to generate the ABTS^+^· stock solution. The stock solution was diluted with phosphate‑buffered saline (pH 7.4) to obtain an absorbance of 0.70 ± 0.02 at 734 nm, which served as the ABTS working solution. For the assay, 1.5 mL of sample solution at various concentrations was mixed with an equal volume of ABTS working solution, vortexed, and incubated in the dark at room temperature for 20 min. The absorbance was measured at 734 nm using ultrapure water as a blank. Two control sets were prepared as described in Section 2.6.1 to obtain A_1_, A_2_, and A_3_. The ABTS radical scavenging activity was calculated using equation (7):

ABTS radical-scavenging activity (%) = [1-(A_1_- A_2_)/A_3_] *100 (7).

A concentration–scavenging rate curve was plotted, and the IC_50_ value was determined.

#### 2.6.3 FRAP reducing capacity

Based on our previously reported method [7], the FRAP assay was used to evaluate the reducing capacity of the samples. The FRAP working solution was freshly prepared by mixing 300 mmol/L acetate buffer (pH 3.6), 10 mmol/L TPTZ solution (in 40 mmol/L HCl), and 20 mmol/L FeCl_3_ solution at a ratio of 10:1:1 (v/v/v). Then, 0.1 mL of sample solution at various concentrations was mixed with 3.0 mL of FRAP working solution, vortexed, and incubated in a water bath at 37 °C for 30 min. The absorbance was measured at 593 nm using ultrapure water as a blank. A standard curve was constructed using Trolox as the reference. The FRAP value was expressed as the sample concentration required to achieve an absorbance of 1.0 value (IC_1.0_) indicates stronger reducing capacity.

#### 2.6.4 Hydroxyl radical scavenging activity

Based on our previously reported method [7], the hydroxyl radical scavenging activity was determined using the salicylic acid method. Then, 1.0 mL of sample solution at various concentrations was sequentially mixed with 1.0 mL of 6 mmol/L FeSO_4_ solution, 1.0 mL of 6 mmol/L salicylic acid‑ethanol solution, and 1.0 mL of 6 mmol/L H_2_O_2_ solution. After vortexing, the mixture was incubated in a water bath at 37 °C for 30 min. The absorbance was measured at 510 nm The hydroxyl radical scavenging activity was calculated using equation (8):

Hydroxyl radical scavenging activity (%) = [1-(A_1_- A_2_)/A_3_] *100 (8).

A concentration–scavenging rate curve was plotted, and the IC_50_ value was calculated.

### 2.7 Characterization

#### 2.7.1 SEM and EDS line scan analysis

A Zeiss Sigma 300 field‑emission SEM was used to examine the surface morphology of raw materials, residues, and extracts from different bio-wastes of *A. manihot* before and after extraction under both UAE and NUAE treatments. Residues were vacuum‑dried, ground, attached to conductive tape, and sputter‑coated with gold for 1 min. Imaging was performed in high‑vacuum mode at 15 kV and a working distance of 10 mm. An Oxford Xplore 300 EDS was used for elemental line scanning to analyze the influence of UAE on surface elemental composition and distribution.

#### 2.7.2 Fourier transform infrared (FTIR) spectroscopy

A Thermo Nicolet iS5 FTIR spectrometer was used to analyze extracts from different bio-wastes of *A. manihot* obtained by UAE. Samples were prepared by the KBr pellet method: 1 mg of dried extract was mixed with 100 mg of spectral‑grade KBr and pressed into a transparent pellet under 10–15 MPa for 30–60 s. Spectra were recorded from 400 to 4000 cm^−1^ at 4 cm^−1^ resolution with 32 scans.

#### 2.7.3 X‑ray diffraction (XRD) analysis

XRD was used to characterize the crystal structure and crystallinity of extracts from different bio-wastes of *A. manihot*. Dried extract powder was evenly packed into the sample holder. XRD patterns were recorded using Cu Kα radiation (λ = 0.15406 nm) at 40 kV and 40 mA, with a 2θ range of 10°–90°, step size 0.02°, and scan speed 2°/min.

#### 2.7.4 Ultraviolet‑visible (UV‑Vis) spectroscopy

A Shimadzu UV-1900 UV‑Vis spectrophotometer was used to record the UV spectra of extracts from different bio-wastes of *A. manihot*. Each dried extract was dissolved in absolute ethanol to 0.5 mg/mL, vortexed, and allowed to stand. Pure ethanol served as the blank. Full‑wavelength scans were performed from 200 to 800 nm at 1 nm intervals. Characteristic absorption peaks were analyzed to compare the composition and content of flavonoids/polyphenols among different plant parts.

### 2.8 UHPLC-MS/MS metabolomics analysis

In this study, untargeted UHPLC-MS/MS metabolomics analysis was performed on 12 samples from different bio-wastes of *A. manihot*. Equal amounts of extracts from each part were accurately weighed, fully dissolved in methanol, and centrifuged at high speed. The supernatant was collected into liquid chromatography vials for analysis. To monitor instrument stability and correct systematic errors, 20 μL of each sample supernatant were mixed to prepare a quality control (QC) pool, which was injected after every four test samples during the run. Chromatographic separation was carried out using a Thermo Scientific Vanquish Horizon UHPLC system equipped with an ACQUITY UPLC HSS T3 column (100 mm × 2.1 mm, 1.8 μm, Waters, USA). The column temperature was maintained at 40 °C, and the injection volume was 3 μL. Mobile phase A consisted of water‑acetonitrile (95:5, v/v) containing 0.1 % formic acid, and mobile phase B consisted of acetonitrile‑isopropanol‑water (47.5:47.5:5, v/v/v) containing 0.1 % formic acid. Mass spectrometry was performed using a UHPLC‑Orbitrap Exploris 240 Fourier‑transform high‑resolution mass spectrometer (Thermo Fisher Scientific, Waltham, MA, USA). Data were acquired in both positive and negative electrospray ionization (ESI^+^/ESI*^−^*) modes. The MS parameters were set as follows: spray voltage 3.4 kV (positive) and 3.0 kV (negative), capillary temperature 320 °C, sheath gas flow 60 arb, auxiliary gas flow 20 arb. Full‑scan MS spectra were recorded in the range of *m*/*z* 70–1050 at a resolution of 60,000, and MS/MS fragmentation was performed at normalized collision energies of 20 %, 40 %, and 60 % at a resolution of 15,000.

Raw MS data were preprocessed using Progenesis QI v3.0 software, including baseline correction, peak detection, retention time alignment, peak integration, and feature quantification. Metabolite annotation was performed by matching against the HMDB, Metlin, and Lipidmaps public databases as well as the Majorbio in‑house metabolite database, using a mass accuracy threshold of < 10 × 10*^−^*^6^ and high similarity of MS/MS fragmentation patterns as criteria. To eliminate experimental errors and improve data accuracy, further data standardization was carried out: metabolite features with > 20 % missing values within any group were removed, remaining missing values were imputed using the global minimum value, spectral response intensities were normalized by the sum normalization method, unstable variables with a relative standard deviation (RSD) > 30 % in QC samples were discarded.

### 2.9 Environmental impact and greenness assessment

The sustainability of the six extraction strategies was comprehensively evaluated based on method-specific energy consumption and multi-parameter greenness metrics. The energy consumption (EC) was calculated as EC = (P × T) / 1000, restricted to the electrical energy directly consumed by the extraction equipment. Furthermore, two greenness assessment tools were employed: AGREEprep (https://mostwiedzy.pl/AGREEprep) evaluated the sample preparation stage based on ten green principles, yielding a standardized score from 0 to 1 with a color-coded pictogram, whereas ComplexGAPI assessed the entire workflow across fifteen categories, grading the overall greenness as excellent (≥75), acceptable (50–74), or inadequate (<50).

### 2.10 Data analysis

All experiments were performed in triplicate, and a significance level of *p* < 0.05 was considered statistically significant. One‑way analysis of variance (ANOVA) was conducted using SPSS 26.0 software (IBM® Corporation, San Jose, CA, USA), followed by Waller‑Duncan multiple range test and Tukey’s multiple comparison test to evaluate the significance of differences among groups. Response surface modeling, regression analysis, and optimization of process parameters were carried out using Design‑Expert 13.0 software (Stat‑Ease® Minneapolis, USA). The construction, training, and performance validation of the BP‑ANN model were performed using the Neural Network Toolbox of MATLAB 2024a software (Portola Valley, CA, USA). In addition, Pearson’s correlation coefficients were calculated to assess the relationships among different indicators, and a correlation heatmap was generated in combination with hierarchical cluster analysis.

## 3 Results and discussion

### 3.1 Ultrasonic system calibration and cavitation quantification

A temperature-controlled UAE platform equipped with a circulating chiller and triple temperature probes was constructed (Fig. 1**A**). The ultrasonic bath was coupled with an external recirculating chiller, and three temperature sensors synchronously monitored the system temperature. The real-time temperature profile (Fig. 1**B**) showed that the system temperature remained within 24.4–25.6 °C with minimal fluctuation, effectively excluding thermal interference and ensuring that sonochemical effects dominated the extraction process. Fig. 1**C** presents the linear calibration between actual ultrasonic intensity and equipment output power, showing an excellent linear relationship (R^2^ = 0.9942). Based on this calibration, a medium acoustic energy density of 113.6 W/L was selected for the extraction experiments to ensure stable cavitation. This quantitative calibration provided a rational basis for selecting acoustic power in subsequent experiments.

Potassium iodide (KI) dosimetry was employed to quantitatively characterize the sonochemical cavitation activity of the ultrasonic system, using triiodide ([*I_3_^−^*]) concentration as a direct measure of cavitation intensity (Fig. 1**D**). At a fixed sonication time of 30 min, triiodide concentration increased significantly with increasing ultrasonic power. At 100 W, essentially no triiodide was generated, whereas at 200–500 W, [*I_3_^−^*] increased continuously, indicating a power threshold for cavitation events [38]. The extraction power of 200 W was selected for this study, and Fig. 1**E** shows the time-course accumulation of triiodide at this power, fitted with both linear and nonlinear models. Both fitting approaches yielded high coefficients of determination, demonstrating that during the entire extraction period, cavitation bubble collapse continuously generated oxidizing reactive species.

### 3.2 Solvent system screening and ultrasound enhancement evaluation

Based on the calibrated acoustic energy input and quantified cavitation activity, three solvent systems and two extraction modes were subsequently compared to identify the optimal UAE system. Three solvent systems (water MS, ethanol MS, and water/ethanol BS, 1:1 v/v) combined with two extraction modes (NUAE and UAE) were applied to four types of *A. manihot* bio-wastes (Fig. 1**F**). TPC and two radical scavenging activities were measured to identify the optimal ultrasound-assisted extraction system (Fig. 2).

**Fig. 2:**
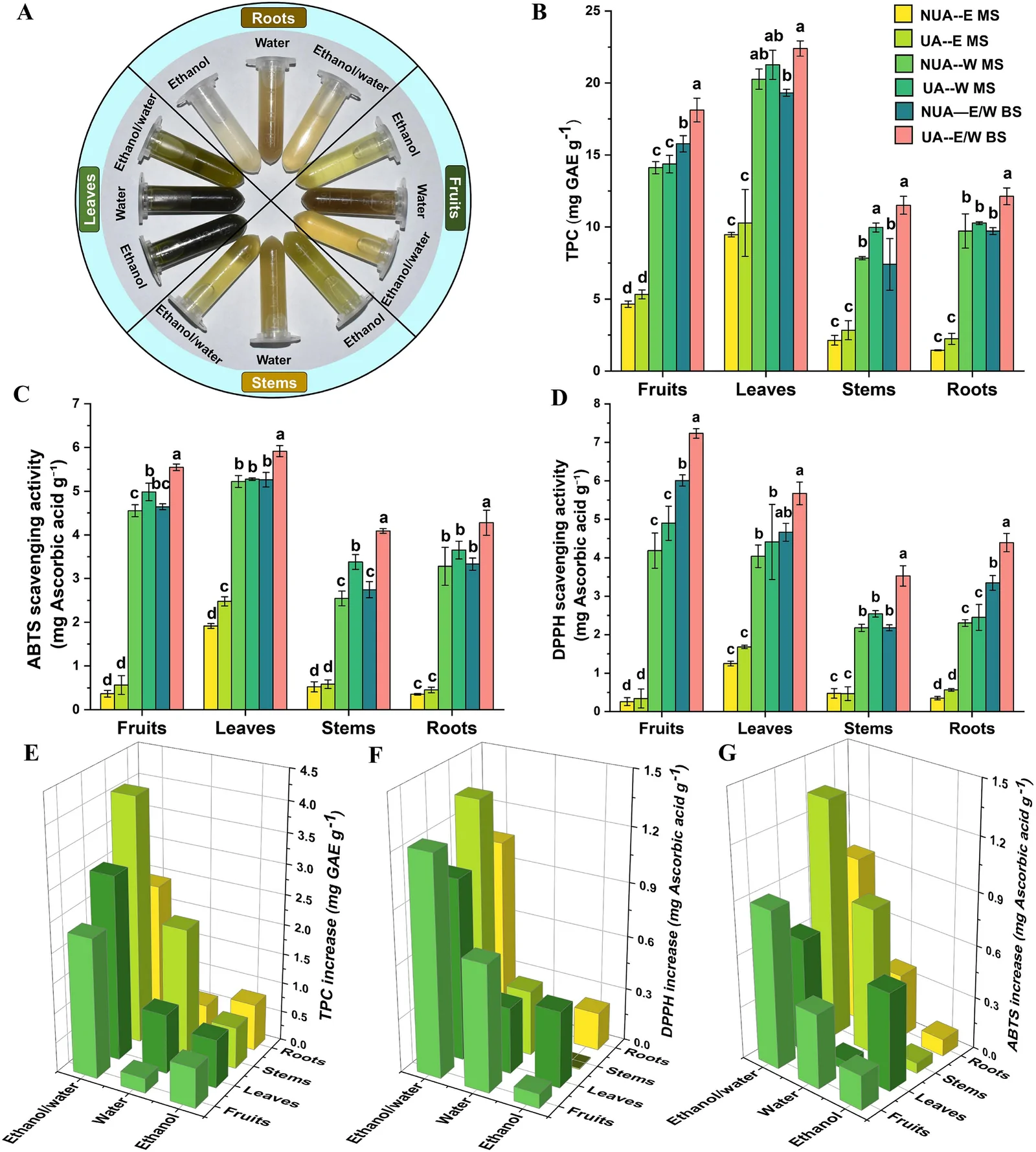
Effects of solvent system and extraction mode on TPC and antioxidant capacity of extracts from different *A. manihot* bio-wastes. (A) Visual appearance of root, stem, leaf and fruit extracts prepared with water, ethanol and water/ethanol (1:1, v/v). (B) TPC. (C) ABTS radical scavenging activity. (D) DPPH radical scavenging activity. (E-G) Net enhancement of TPC (E), DPPH (F) and ABTS scavenging activity (G) for UAE compared with NUAE across different solvents and plant matrices. Different lowercase letters indicate significant differences (*p* < 0.05).

The extract appearance (Fig. 2**A**) provided preliminary visual evidence of ultrasound-solvent synergy. UA-E/W BS extracts from fruit and leaf showed dark brown-green coloration, while mono-solvent extracts appeared pale yellow. This color deepening was attributed to ultrasound-promoted co-release of chlorophyll, flavonols, and phenolic acids, consistent with our previous findings on *A. manihot* flower extraction [6]. From a process perspective, ultrasound treatment enhanced convective mass transfer of solvent into disrupted cellular compartments, facilitating the simultaneous release of polar (phenolic acids) and medium-polar (flavonoids) phytochemicals that collectively contributed to the dark pigmentation [45]. Quantitative TPC data (Fig. 2**B**) confirmed that UA-E/W BS achieved significantly higher equilibrium polyphenol yields than all mono-solvent and non-sonicated groups across all four tissue matrices (*p* < 0.05). For leaf waste, TPC reached 22.13 mg GAE g^−1^ under UA-E/W BS, with a net ultrasound-induced increment of 3.87 mg GAE g^−1^ relative to NUAE under identical binary solvent conditions (Fig. 2**E**). Pure ethanol produced the lowest TPC regardless of ultrasound application, because its high polarity failed to extract the abundant medium-polar phenolic compounds constituting the major polyphenol pool in *A. manihot* [46]. Pure water also showed lower extraction capacity than the binary solvent, possibly due to protein denaturation and matrix shrinkage that reduced solvent accessibility to intracellular polyphenols [47]. These results confirmed that the coupling of ultrasound treatment with water–ethanol polarity matching was indispensable for efficient polyphenol recovery. Antioxidant activity trends (Fig. 2**C-D**) were highly consistent with TPC variation. UA-E/W BS exhibited the strongest ABTS and DPPH radical scavenging capacity across all four tissues. For leaf samples, ABTS and DPPH activities reached 6.12 and 7.34 mg ascorbic acid g^−1^, respectively, significantly higher than other extraction treatments (*p* < 0.05). The three-dimensional net enhancement plots (Fig. 2**E-G**) further quantified the ultrasound intensification amplitude. The water/ethanol binary solvent yielded much larger increments of TPC, DPPH, and ABTS activities under UAE than mono-solvents, and the ultrasound-induced enhancement of antioxidant activity positively correlated with elevated polyphenol concentration. Notably, short-duration controlled UAE (30 min, calibrated acoustic power 200 W) effectively avoided polyphenol oxidative degradation. Although ultrasound introduced localized energy input, the treatment substantially shortened the extraction equilibrium time and reduced prolonged exposure of polyphenols to oxygen and thermal stress [48]. Moreover, the binary solvent selectively dissolved high-activity medium-polar polyphenolic fractions [6], further enriching the antioxidant potency of extracts.

The physical effects of ultrasound in liquid–solid systems, including micro-streaming and pressure fluctuations, can exert mechanical actions on cell walls [40], [41]. As schematically illustrated in Fig. 1**F**, this physical perturbation, combined with solvent polarity regulation, generated a synergistic effect that promoted polyphenol release. The water/ethanol binary system offered unique advantages: water penetrated plant tissue to induce cell wall swelling and loosen lignocellulosic crosslinking *via* hydrogen-bond disruption [42], while ethanol dissolved medium-polar flavonoids and phenolic acids. Pure water, despite its swelling capacity, could not effectively solubilize medium-polar phenolic compounds due to its high dielectric constant [43]. Anhydrous ethanol failed to swell the lignocellulosic matrix because it lacked the hydrogen-bonding capacity to disrupt polysaccharide networks [44]. Fruit and leaf matrices exhibited the most pronounced ultrasound enhancement, originating from their thin cell walls and low lignification degree, which made them more susceptible to ultrasound mechanical effects. In contrast, highly lignified stem and root tissues showed weaker responses, revealing strong matrix-ultrasound interactions that will be further elucidated through kinetic and microstructural analyses in the following sections.

### 3.3 Ultrasound-dependent extraction kinetic modeling and mass transfer mechanism

Fig. 3 presents the polyphenol extraction kinetic curves for four *A. manihot* bio-wastes under different extraction modes and solvent systems, fitted with pseudo-first-order and pseudo-second-order kinetic models. Fig. 3**A-L** show the TPC kinetic profiles, the linear fitting plots of the pseudo-first-order model, and the linear fitting plots of the pseudo-second-order model. The corresponding kinetic parameters are compiled in Table 1 and Supplementary Table 2. The pseudo-second-order kinetic model provided superior fitting performance in nearly all treatment groups. Under linear regression, the pseudo-second-order model yielded R^2^ values mostly above 0.99, significantly more stable than the pseudo-first-order model. Under nonlinear fitting, the pseudo-second-order model also achieved higher R^2^ values, and the predicted theoretical equilibrium polyphenol yields more closely matched the experimentally observed plateau values. This indicated that polyphenol extraction under both UAE and NUAE followed chemisorption-controlled mass transfer kinetics rather than simple diffusion-limited processes. This finding is consistent with the mechanism that polyphenol dissolution involves adsorption/desorption equilibrium at the solid–liquid interface, where the rate-determining step is the affinity between solute molecules and the solvent rather than solely molecular diffusion [49], [50]. These results explain the solvent-dependent extraction efficiency observed in Section 3.2. Specific model fitting comparison further verified the superiority of the pseudo‑second‑order kinetic model for describing the present extraction process. Specifically, for fruit tissue under UA‑E/W BS, the pseudo‑second‑order model yielded a higher coefficient of determination (R^2^ = 0.9895) and a larger theoretical equilibrium TPC (*Y_TPC_* = 21.49 mg GAE g^−1^), compared with the pseudo‑first‑order fitting results (R^2^ = 0.9769), *Y_TPC_* = 18.35 mg GAE g^−1^). Consistent results were observed in leaf tissue: the pseudo‑second‑order model for UA‑E/W BS produced an R^2^ of 0.9818 and a *Y_TPC_* of 28.58 mg GAE g^−1^, which outperformed the pseudo‑first‑order fitting. The kinetic rate constant *k_2_* of all UAE groups was distinctly higher than matched NUAE treatments under the same solvent. This directly demonstrated that ultrasound treatment reduced the mass transfer resistance of polyphenols across cell wall barriers. Among all experimental groups, UA-E/W BS exhibited the maximum *k_2_* and theoretical equilibrium TPC for fruit, leaf, stem, and root, providing numerical kinetic evidence for the optimal ultrasound-solvent combination identified in the screening experiments above. For leaf tissue, the *k_2_* of UA-E/W BS was 0.0044 g·mg^−1^·min^−1^, approximately 1.4-fold higher than NUA-E/W BS (0.0031). This 1.2- to 1.5-fold enhancement magnitude is highly consistent with recent kinetic studies on ultrasound-assisted extraction of lignocellulosic plant materials [51], confirming that the observed ultrasound-induced enhancement is a typical and robust phenomenon. Therefore, for leaf tissue, ultrasound may effectively shorten the equilibrium time by disrupting lignocellulosic structures. From a process perspective, the physical perturbations generated by ultrasound propagation in the medium, including micro-streaming effects and associated pressure variations, collectively enhanced the convective mass transfer coefficient. This was directly reflected in the elevated kinetic constants [52], [51].

**Fig. 3:**
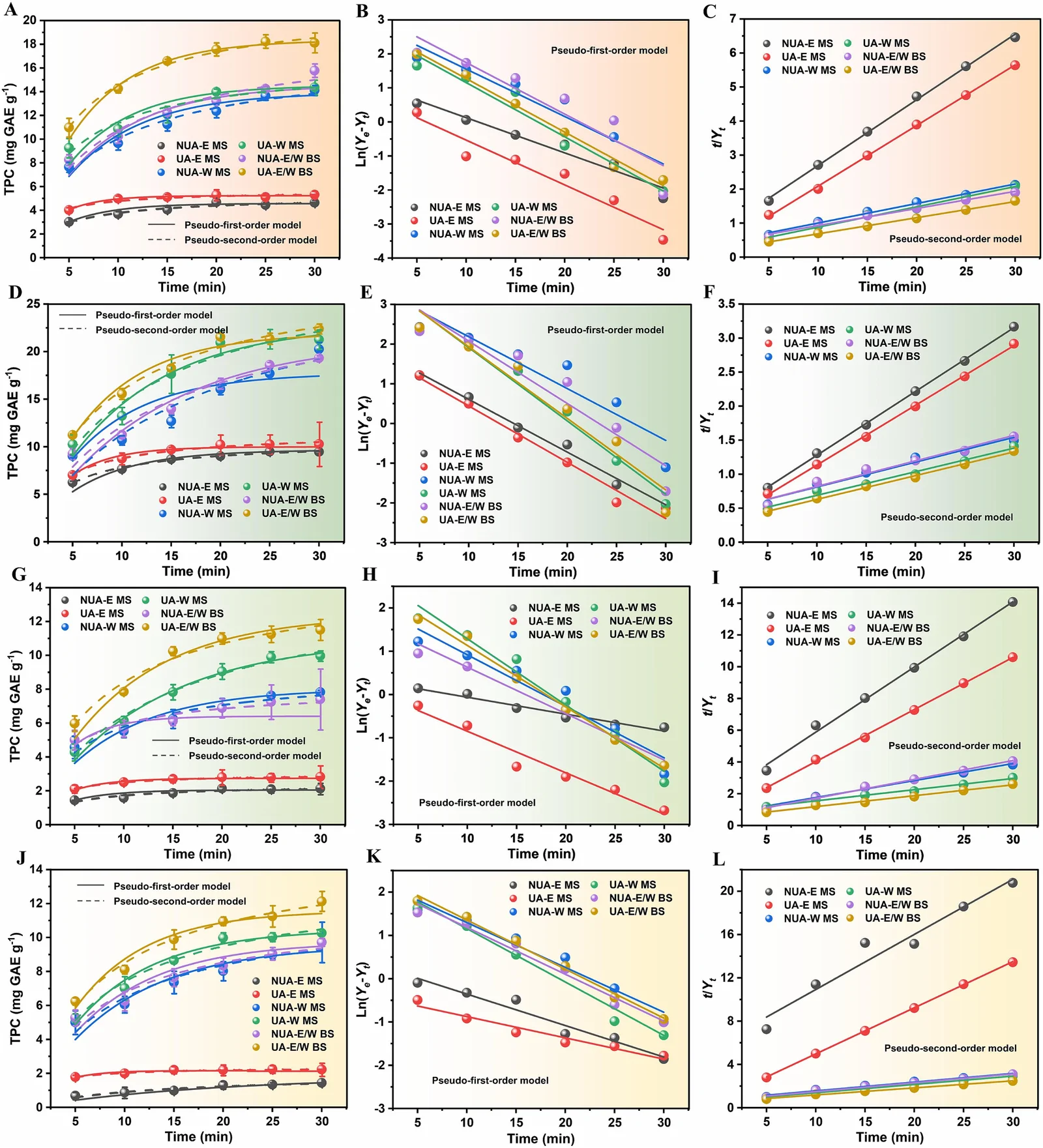
TPC extraction kinetic curves of four *A. manihot* bio-wastes under different extraction modes and solvent systems, fitted with pseudo-first-order and pseudo-second-order kinetic models. (A, D, G, J) TPC kinetic profiles; (B, E, H, K) linear fitting plots of pseudo-first-order model; (C, F, I, L) linear fitting plots of pseudo-second-order model. (A-C) Fruit; (D-F) Leaf; (G-I) Stem; (J-L) Root.

**Table 1:** Fitting parameters of pseudo-first-order and pseudo-second-order kinetic models for total polyphenol extraction from *A. manihot* bio-wastes *via* nonlinear and linear regression under different extraction conditions.

Part	System	Non-linear form						linear form					
		Pseudo-first order			Pseudo-second order			Pseudo-first order			Pseudo-second order		
		*k_1_* (min^−1^)	*Y_TPC_* (mg/g)	R^2^	*k_2_* (g·mg^−1^·min^−1^)	*Y_TPC_* (mg/g)	R^2^	*k_1_* (min^−1^)	*Y_TPC_* (mg/g)	R^2^	*k_2_* (g·mg^−1^·min^−1^)	*Y_TPC_* (mg/g)	R^2^
Fruit	NUA-E MS	0.2151	4.5679	0.9706	0.0488	5.2656	0.9454	0.1034	3.1852	0.9582	0.0486	5.1843	0.9984
	UA-E MS	0.2953	5.2247	0.9854	0.0937	5.6972	0.9203	0.1316	2.1866	0.9387	0.1037	5.6148	0.9993
	NUA-W MS	0.1355	13.9281	0.9399	0.0083	17.0984	0.9722	0.1394	19.0642	0.9264	0.0077	17.4490	0.9929
	UA-W MS	0.1552	14.5271	0.9010	0.0137	16.4781	0.9405	0.1593	15.6297	0.9606	0.0124	16.7729	0.9935
	NUA-E/W BS	0.1302	14.5676	0.8966	0.0072	18.7012	0.9548	0.1514	26.0217	0.8652	0.0060	19.8570	0.9906
	UA-E/W BS	0.1564	18.3504	0.9769	0.0097	21.4930	0.9895	0.1573	17.2344	0.9900	0.0107	21.1015	0.9983
Leaf	NUA-E MS	0.1576	9.6571	0.9927	0.0254	10.7188	0.9905	0.1328	6.9225	0.9909	0.0259	10.6633	0.9995
	UA-E MS	0.2408	9.9838	0.9915	0.0270	11.6102	0.9865	0.1418	6.4160	0.9804	0.0301	11.3934	0.9993
	NUA-W MS	0.1353	17.7240	0.8845	0.0021	29.4080	0.9127	0.1306	32.8099	0.8704	0.0030	27.4499	0.9737
	UA-W MS	0.0999	22.8721	0.9578	0.0032	29.8238	0.9569	0.1849	43.0176	0.9708	0.0035	28.8434	0.9853
	NUA-E/W BS	0.0792	21.3174	0.9648	0.0031	26.7257	0.9527	0.1570	38.2506	0.8999	0.0032	26.6738	0.9693
	UA-E/W BS	0.1381	21.9596	0.9776	0.0044	28.5837	0.9818	0.1804	41.9232	0.9454	0.0043	28.6697	0.9969
Stem	NUA-E MS	0.2443	2.0450	0.9134	0.1048	2.4211	0.8970	0.0391	1.3879	0.9641	0.0951	2.4374	0.9953
	UA-E MS	0.2734	2.7338	0.9232	0.1504	3.0644	0.9876	0.0959	1.1161	0.9597	0.1461	3.0484	0.9993
	NUA-W MS	0.1198	8.0301	0.9473	0.0190	9.1094	0.9477	0.1192	8.2337	0.9378	0.0167	9.3449	0.9916
	UA-W MS	0.0852	10.9699	0.9892	0.0055	14.4913	0.9903	0.1533	16.7821	0.9754	0.0059	14.2066	0.9914
	NUA-E/W BS	0.2631	6.4038	0.6425	0.0345	8.0870	0.9130	0.1074	5.5023	0.9681	0.0270	8.4189	0.9934
	UA-E/W BS	0.1048	12.4015	0.9522	0.0090	14.7136	0.9725	0.1420	12.8404	0.9919	0.0094	14.5858	0.9915
Root	NUA-E MS	0.0519	1.8298	0.9860	0.0443	1.9647	0.9297	0.0728	1.4517	0.9540	0.0445	1.9646	0.9541
	UA-E MS	0.3361	2.1562	0.8407	0.2462	2.3913	0.9584	0.0491	0.6802	0.9500	0.2595	2.3454	0.9999
	NUA-W MS	0.1079	9.5618	0.9337	0.0098	11.9401	0.9453	0.1036	10.3058	0.9623	0.0087	12.3977	0.9833
	UA-W MS	0.1248	10.4960	0.9834	0.0095	13.2680	0.9779	0.1234	10.9290	0.9669	0.0100	12.9282	0.9965
	NUA-E/W BS	0.1191	9.7548	0.9153	0.0119	11.6247	0.9490	0.1083	9.6749	0.9770	0.0100	12.2504	0.9875
	UA-E/W BS	0.1399	11.5819	0.9575	0.0089	14.9195	0.9858	0.1134	12.0645	0.9926	0.0081	15.3304	0.9968

*k_1_*: rate constant of pseudo–first–order model; *Y_TPC_*: theoretical equilibrium total polyphenol content; *R^2^*: coefficient of determination; *k_2_*: rate constant of pseudo–second–order model.

Notable differences were observed between linear and nonlinear fitting in predicting equilibrium yields. For example, in the leaf pure water UAE group (UA-W MS), the pseudo-first-order linear fitting gave a *Y_TPC_* of 43.0176 mg GAE g^−1^, which deviated significantly from the nonlinear fitting value of 22.8721 mg GAE g^−1^ and the experimental data. In contrast, the pseudo-second-order model showed good consistency between linear and nonlinear fitting (R^2^ > 0.98). This discrepancy arises from error accumulation during the logarithmic transformation in the pseudo-first-order linearization, whereas the pseudo-second-order model, which does not involve such transformation, demonstrates excellent robustness and provides more reliable equilibrium predictions for describing polyphenol UAE kinetics.

Matrix-dependent kinetic differences were clearly observed. Under identical ultrasound parameters, fruit and leaf showed higher rate constants and equilibrium TPC than stem and root. For instance, under UA-E/W BS, the pseudo-second-order *k_2_* values for fruit, leaf, stem, and root were 0.0097, 0.0044, 0.0090, and 0.0089 g·mg*^−^*^1^·min*^−^*^1^, respectively. The corresponding theoretical *Y_TPC_* values were 21.49, 28.58, 14.71, and 14.92 mg GAE g^−1^. These results reflected that dense lignified fiber bundles in stem and root more significantly hindered solvent penetration and solute diffusion [7]. Under mono-solvent systems, the gap of *k_2_* between UAE and NUAE narrowed considerably. For example, stem NUA-E MS gave a *k_2_* of 0.1048, while UA-E MS yielded 0.1504, a modest increase compared to the dramatic enhancement observed in binary solvent systems. This further confirmed that the water/ethanol binary solvent, by pre-conditioning the cell wall structure, synergistically amplified the ultrasound-induced mass transfer promotion. This synergistic effect can be explained as follows: sufficient solvent penetration into the cell wall matrix is a prerequisite for effective ultrasound action. Without adequate swelling, the input energy would be largely dissipated in the rigid lignocellulosic framework rather than effectively promoting disruption and mass transfer [53]. The binary solvent thus served a dual function. It chemically weakened the cell wall structure through hydration and hydrogen-bond disruption, while simultaneously providing a polarity-matched environment for solubilizing the released polyphenols, thereby maximizing the extraction efficiency per unit energy input (as conceptually depicted in Fig. 1**F**). It is also noteworthy that for some mono-solvent systems, especially under non-ultrasonic conditions, the pseudo-first-order model occasionally gave higher R^2^ values than the pseudo-second-order model (fruit NUA-W MS: pseudo-first-order R^2^ = 0.9399 *vs.* pseudo-second-order R^2^ = 0.9722; root UA-E MS: pseudo-first-order R^2^ = 0.8407 *vs*. pseudo-second-order R^2^ = 0.9584). However, the pseudo-second-order model consistently provided more realistic equilibrium predictions that better matched the experimentally observed plateau values, confirming its overall superiority for describing the extraction kinetics of polyphenols from lignocellulosic matrices.

To establish a quantitative link between cavitation intensity and polyphenol extraction kinetics, linear correlation analysis was performed for the pure water mono-solvent UAE system (Fig. 4**A, B**). The equilibrium polyphenol yield (*Y*_TPC_) of the four bio-wastes exhibited strong linear correlations with steady-state [*I_3_^−^*], with R^2^ > 0.97 for all tissues (Fig. 4**A**). The polyphenol extraction rate (d*Y*_TPC_/d*t*) also showed a positive linear relationship with the triiodide formation rate (d[*I_3_^−^*]/d*t*), although the fitting R^2^ was lower than that for equilibrium yields, with the fruit group showing the most pronounced deviation (Fig. 4**B**). These results demonstrate that higher steady-state cavitation activity corresponds to higher equilibrium polyphenol recovery, and that the conversion of cavitation effects into solid–liquid mass transfer acceleration exhibits tissue-specific differences.

**Fig. 4:**
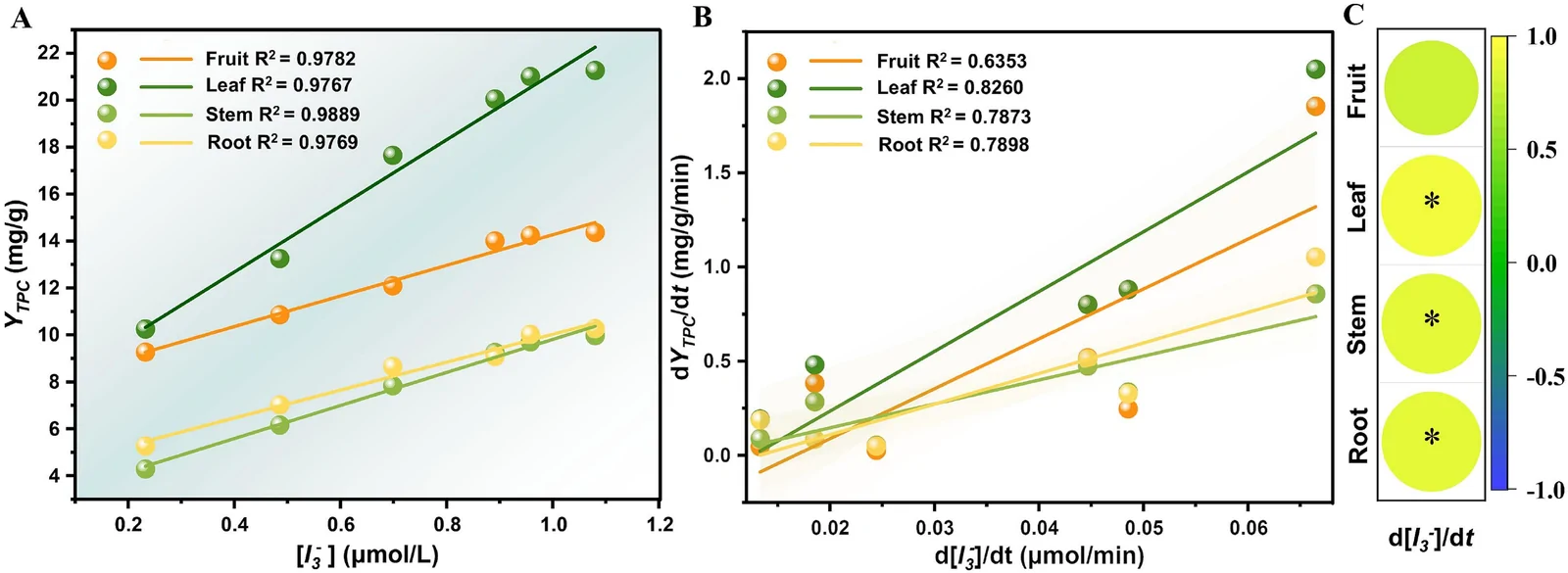
Correlation between triiodide ([*I_3_^−^*]) generation and TPC under UA-W MS system from four *A. manihot* bio-wastes. (A) Linear fitting between equilibrium TPC yield (*Y_TPC_*) and steady-state [*I_3_^−^*]; (B) Linear fitting between the TPC extraction rate (dY*_TPC_*/d*t*) and triiodide formation rate (d[*I_3_^−^*]/d*t*); (C) Correlation bubble plot between d[*I_3_^−^*]/d*t* and dY*_TPC_*/d*t*.

### 3.4 Microstructural visualization of ultrasound-induced cell wall disruption

SEM (Fig. 5) provided direct microscale morphological evidence to interpret the physical effects of ultrasound on plant tissues. Intact raw powder, UAE-treated residues, and conventional NUAE residues from four agro-waste matrices were compared.

**Fig. 5:**
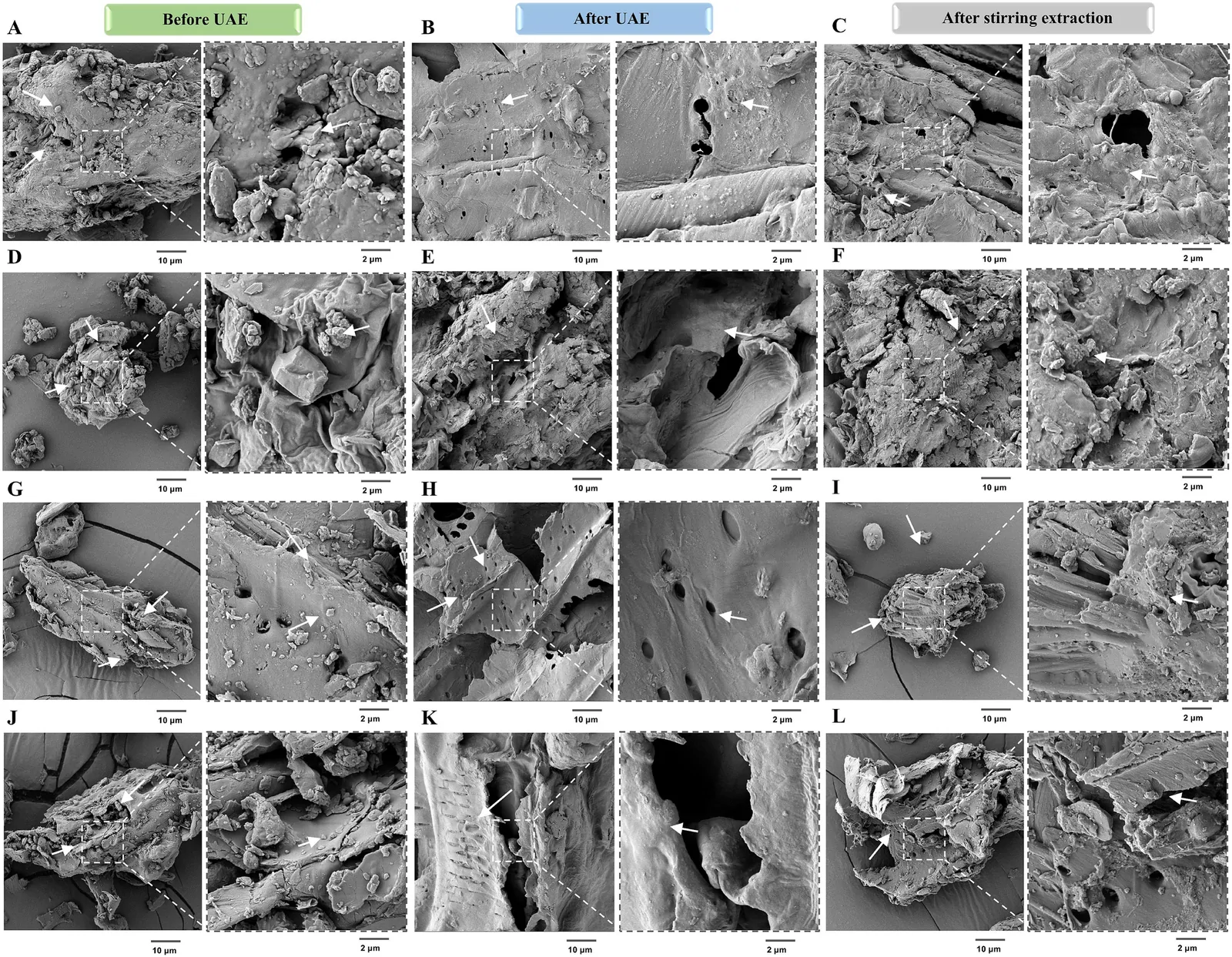
Comparative effects of UAE and NUAE on the microstructure of powder samples from *A. manihot* bio-wastes. (A–C) Fruit, (D–F) leaf, (G–I) stem, (J–L) root; panels show morphology before extraction, after UAE and after NUAE, respectively. Scale bars: 10 μm and 2 μm.

Untreated fruit (Fig. 5**A**), leaf (Fig. 5**D**), stem (Fig. 5**G**), and root (Fig. 5**J**) powders exhibited compact, smooth, and continuous surface structures with negligible pores or cracks. The intact lignocellulosic cell walls may act as a dense physical barrier that blocked polyphenol outward diffusion, which is further supported by our kinetic results. After conventional shake-assisted extraction (Fig. 5**C, 5F, 5I, and 5 L**), only minor superficial peeling appeared on tissue surfaces, and the cell wall frameworks remained largely intact. This morphological observation is consistent with the low equilibrium TPC and slow mass transfer rates of NUAE groups observed in kinetic analysis (Section 3.3). The lack of obvious structural disruption under NUAE suggests that passive diffusion alone was insufficient to overcome the intrinsic resistance imposed by the lignocellulosic matrix, as quantified by our kinetic modelling [54]. In contrast, UAE-treated samples exhibited significant microstructural changes. Fruit tissue surfaces developed abundant deep holes and concave depressions (Fig. 5**B**). Leaf matrices were torn into loose, wrinkled porous fragments with interconnected large cavities (Fig. 5**E**). Stem lamellar fiber structures split and generated wide penetrating cracks (Fig. 5**H**). Dense fibrous bundles of root fractured and peeled off, with markedly enlarged intercellular voids (Fig. 5**K**). These morphological alterations provided direct visual evidence for the ultrasound-induced cell wall disruption mechanism schematically depicted in Fig. 1**F**. Among all tissues, leaf and fruit showed the most severe structural changes, which is consistent with their largest ultrasound-induced TPC and antioxidant increments in Section 3.2 and their highest kinetic mass transfer constants in Table 1. The severity of microstructural disruption directly correlated with extraction efficiency enhancement, and the combination of SEM morphological observations and kinetic fitting jointly supports the link between ultrasound treatment, cell wall structural damage, and polyphenol release [55]. Fruit and leaf contained thin primary cell walls with low cellulose and lignin content, making them more susceptible to structural changes under ultrasound treatment. Stem and root were rich in lignin and cellulose, forming rigid compact fiber networks that exhibited stronger resistance to ultrasound physical effects. Consequently, the absolute amount of polyphenols released from stem and root was limited even under identical calibrated ultrasound intensity. This composition-dependent response has been previously reported for lignocellulosic biomass subjected to ultrasound treatment, where the physicochemical properties of the solid matrix and their interaction with the liquid phase determine the efficiency of energy transfer toward structural disruption [56], [57]. For highly lignified tissues, a substantial portion of the input energy was likely absorbed by the rigid fiber network without inducing significant structural changes, resulting in lower extraction yields. This structural-mechanical rationale also explained why the pseudo-second-order rate constants for stem and root remained substantially lower than those for leaf and fruit across all solvent systems (Table 1). Denser matrices required higher energy input to achieve comparable levels of cell wall disruption.

Collectively, SEM observations provide visual evidence of ultrasound-induced physical disruption of plant lignocellulosic barriers at the tissue surface. Such surface morphological changes may generate potential mass transfer channels and increase tissue specific surface area. The kinetic fitting results independently quantify the acceleration of intracellular polyphenol dissolution and diffusion. The combination of SEM micrographs and kinetic data supports the ultrasound‑solvent synergistic effect, which governs the final polyphenol extraction efficiency from *A. manihot* bio-wastes.

### 3.5 Optimization of UAE process parameters

#### 3.5.1 Single-factor experiments of UAE process

Following solvent and extraction mode screening, single-factor trials were implemented to clarify how ethanol proportion, ultrasonic duration, LSR and ultrasonic power affect TPC, DPPH and ABTS activity of four *A. manihot* bio-wastes, with varied response curves summarized in Fig. 6**.**

**Fig. 6:**
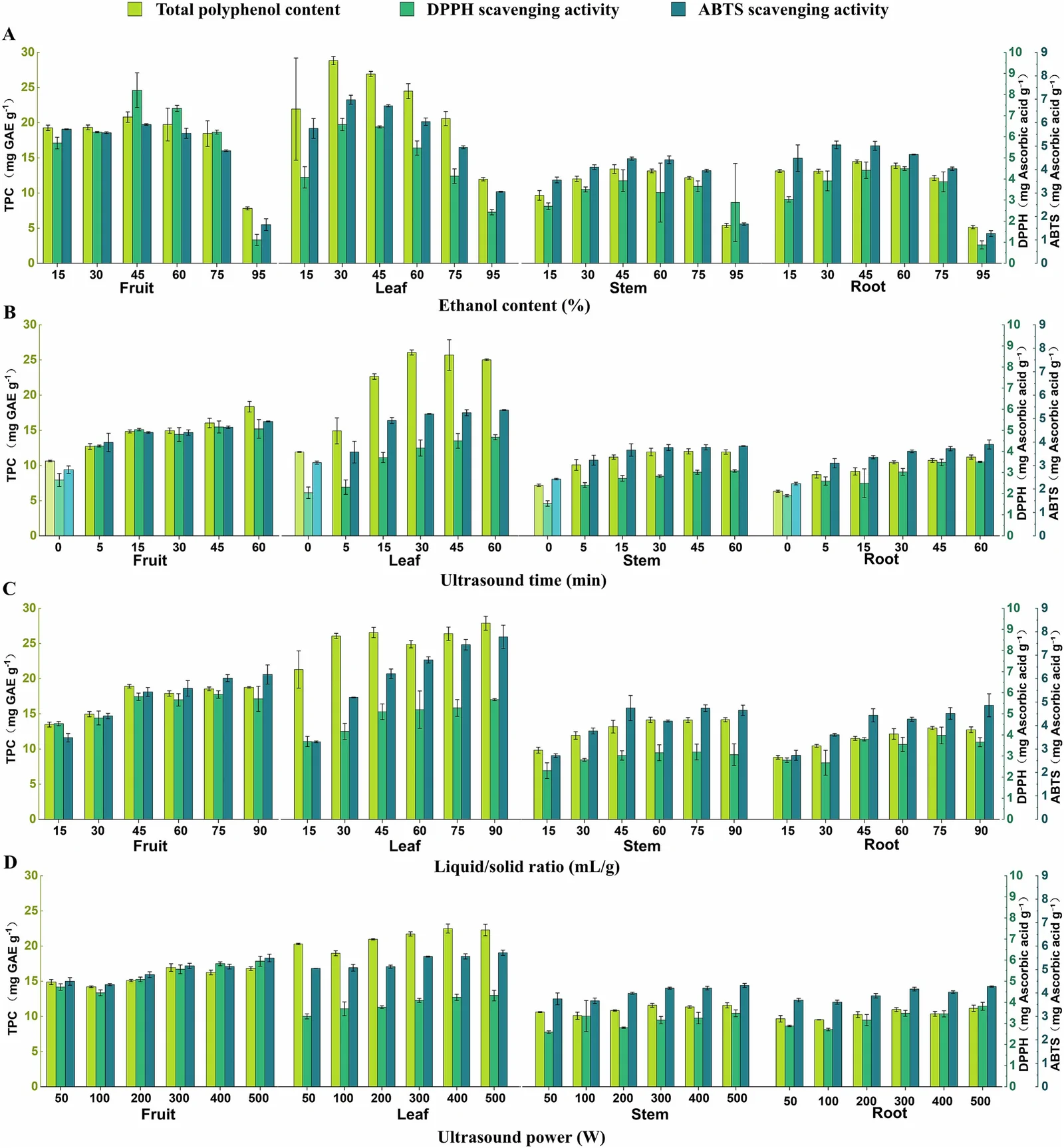
Single-factor optimization of UAE parameters for TPC and antioxidant activities from different bio-wastes of *A. manihot*. (A) Effect of ethanol content (15 – 95 %, v/v). (B) Effect of ultrasound time (0 – 60 min). (C) Effect of LSR (15 – 90 mL/g). (D) Effect of ultrasonic power (50 – 500 W). Data are presented as mean ± standard deviation (n = 3).

Ethanol content determines solvent polarity and solubility of phenolic compounds. As shown in Fig. 6**A**, all samples exhibited a rise-then-fall unimodal trend with increasing ethanol fraction, while the peak yields and optimal concentrations varied among the four tissues. Leaves achieved maximum TPC (28.5 mg GAE·g^−1^) at 45 % ethanol, 68.2 % higher than 15 % group, alongside peak antioxidant indexes, yet dropped sharply to merely 31.2 % of maximal value at 95 % ethanol. Fruit TPC topped at 30 – 45 % ethanol (20.2 mg GAE·g^−1^), with mild attenuation at high ethanol. Stem peaked at 45 % (10.8 mg GAE·g^−1^, only 37.9 % of leaf’s peak) and fluctuated moderately, root optimal point shifted to 60 % ethanol (10.2 mg GAE·g^−1^) with flat variation. Such divergence originated from phenolic polarity discrepancy, leaf and fruit accumulate medium-polar flavonoid glycosides best soluble in 40 – 50 % aqueous ethanol, while root phenolics are dominated by low-polar lipophilic constituents favoring higher ethanol ratio. Extreme solvent polarity either fails to dissolve target phenolics or suppresses matrix hydration and triggers phenolic oxidation, and abundant active flavonoids in leaf and fruit are vulnerable to high ethanol [39], [41]. Hence 15 %, 45 %, 75 % ethanol were fixed for RSM design.

Ultrasonic time critically influences cavitation intensity, solvent penetration, and mass transfer, but excessive sonication may induce thermal or oxidative degradation of polyphenols. As shown in Fig. 6**B**, TPC and antioxidant capacity first increased and then decreased with prolonged ultrasonic time. Leaf peaked at 30 – 45 min (26.8 mg GAE·g^−1^ at 45 min) and decreased 27.2 % at 60 min, fruit maximum appeared at 30 min (18.5 mg GAE·g^−1^) with a mild 18.9 % drop at 60 min. Stem and root hit their maxima at 45 min and 30 – 45 min respectively, with lower yield increments and weaker post-peak degradation. Short sonication facilitates cell rupture and phenolic outflow, whereas prolonged treatment elevates local temperature and free radical content to induce phenolic oxidation and polymerization [48], [49], [58]. Unstable flavonoid glycosides in leaf and fruit degrade more readily than phenolics in lignified stem and root. Ultrasonic durations of 5, 30 and 55 min were chosen for subsequent optimization.

LSR critically affects solvent–matrix contact, solute diffusion, and cavitation energy distribution. As shown in Fig. 6**C**, polyphenol yield first increased with rising LSR, then reached a plateau or underwent a slight reduction in all four tissues. Leaf TPC maximized at 60 – 75 mL/g (27.5 mg GAE·g^−1^) and fell marginally above 75 mL/g, fruit stabilized after peaking at 45 – 60 mL/g, stem optimum occurred at 60 mL/g, while root required 75 – 90 mL/g to reach the highest yield without obvious decline. Thin-walled leaf and fruit achieve full phenolic release at moderate solvent dosage, whereas compact lignified stem and root demand higher LSR for sufficient solvent infiltration. Too low LSR leads to incomplete sample wetting, while excess solvent dilutes ultrasonic energy and raises downstream concentration cost [59], [60]. LSR of 10, 45 and 80 mL/g were selected to balance efficiency and cost.

Ultrasonic power governs cavitation intensity and mass transfer efficiency. As shown in Fig. 6**D**, the extraction yield first rose and then fell with increasing power, accompanied by marked differences in tissue sensitivity among the four parts. Leaf and fruit obtained optimal extraction at 200 – 300 W, yet leaf underwent a steeper post-peak reduction (29.4 % at 500 W) than fruit (17.6 %). Stem peaked at 300 W and root at 300 – 400 W, both showing milder yield loss at high power. Moderate power strengthens cavitation shear force to disrupt cell wall, excessive power causes thermal spike and radical accumulation to decompose heat-sensitive flavonoids in leaf and fruit [24], [28]. Therefore 300 W was set as the central power for RSM.

Overall, four parameters significantly affect phenolic extraction, and leaf/fruit exhibit higher parameter sensitivity due to loose tissue and high active flavonoid content, in contrast to wide-adaptability of stem and root. The screened variable ranges lay solid foundation for subsequent response surface modeling.

#### 3.5.2 Determination of optimal extraction conditions

Based on the effective parameter ranges determined by single-factor experiments, a quadratic regression model was established using Box-Behnken design (BBD) to optimize the UAE parameters, ethanol content (A), ultrasonic time (B), and LSR (C), for polyphenol extraction from fruit, leaf, stem, and root bio-wastes of *A. manihot*. Coded levels and actual values are listed in Supplementary Table 3, and the complete experimental design matrix with observed responses for TPC, DPPH, and ABTS is compiled in Supplementary Table 4. ANOVA results (Supplementary Tables 5–8) confirmed that the quadratic models for all four tissues were statistically robust, with highly significant F-values (*p* < 0.0001) and non-significant lack-of-fit (*p* > 0.05). For fruit tissue, the main-effect ranking was C > B > A, with C and B being highly significant (*p* < 0.0001). For leaf, stem, and root tissues, C was the most critical factor, followed by A and B. The significance levels of interaction terms varied among tissues. All quadratic coefficients were negative and significant across the four tissues, and the convex response surfaces (Fig. 7**A–L**; Supplementary Figs. 2–5) confirmed the existence of optimal extraction ranges. Ultrasonic cavitation disrupted cell walls of different tissues to varying degrees, with thin leaf cell walls being more easily disrupted than the highly lignified stem and fibrous root tissues [23], [61], as reflected in the distinct response patterns and optimal parameter ranges. The BBD cube plots (Supplementary Fig. 6) and corresponding contour plots (Supplementary Figs. 7–10) further illustrated the interactive effects of the three variables on TPC, DPPH, and ABTS. Box-Cox transformation diagnostics (Supplementary Fig. 11) confirmed that the optimal lambda values for all response variables lay within the 95 % confidence interval and that the model residuals satisfied normality assumptions. Observed-versus-predicted scatter plots (Supplementary Fig. 12) showed that data points clustered tightly along the diagonal line with very low dispersion, directly validating the strong agreement between model predictions and experimental results. Through multi-objective optimization, the optimal UAE conditions were determined as follows: for fruit, A = 43.62 %, B = 36.85 min, C = 57.21 mL/g, with predicted TPC, DPPH, and ABTS values of 25.26, 8.35, and 6.89 mg/g, respectively; for leaf, A = 44.17 %, B = 35.29 min, C = 61.35 mL/g, with predicted values of 28.93, 5.12, and 8.11 mg/g; for stem, A = 42.35 %, B = 33.72 min, C = 59.46 mL/g, with predicted values of 16.72, 4.29, and 5.27 mg/g; and for root, A = 41.88 %, B = 34.61 min, C = 58.73 mL/g, with predicted values of 13.92, 4.89, and 5.42 mg/g. Validation experiments under these optimal conditions yielded errors below 3 % for all tissues, confirming the reliability of the established models.

**Fig. 7:**
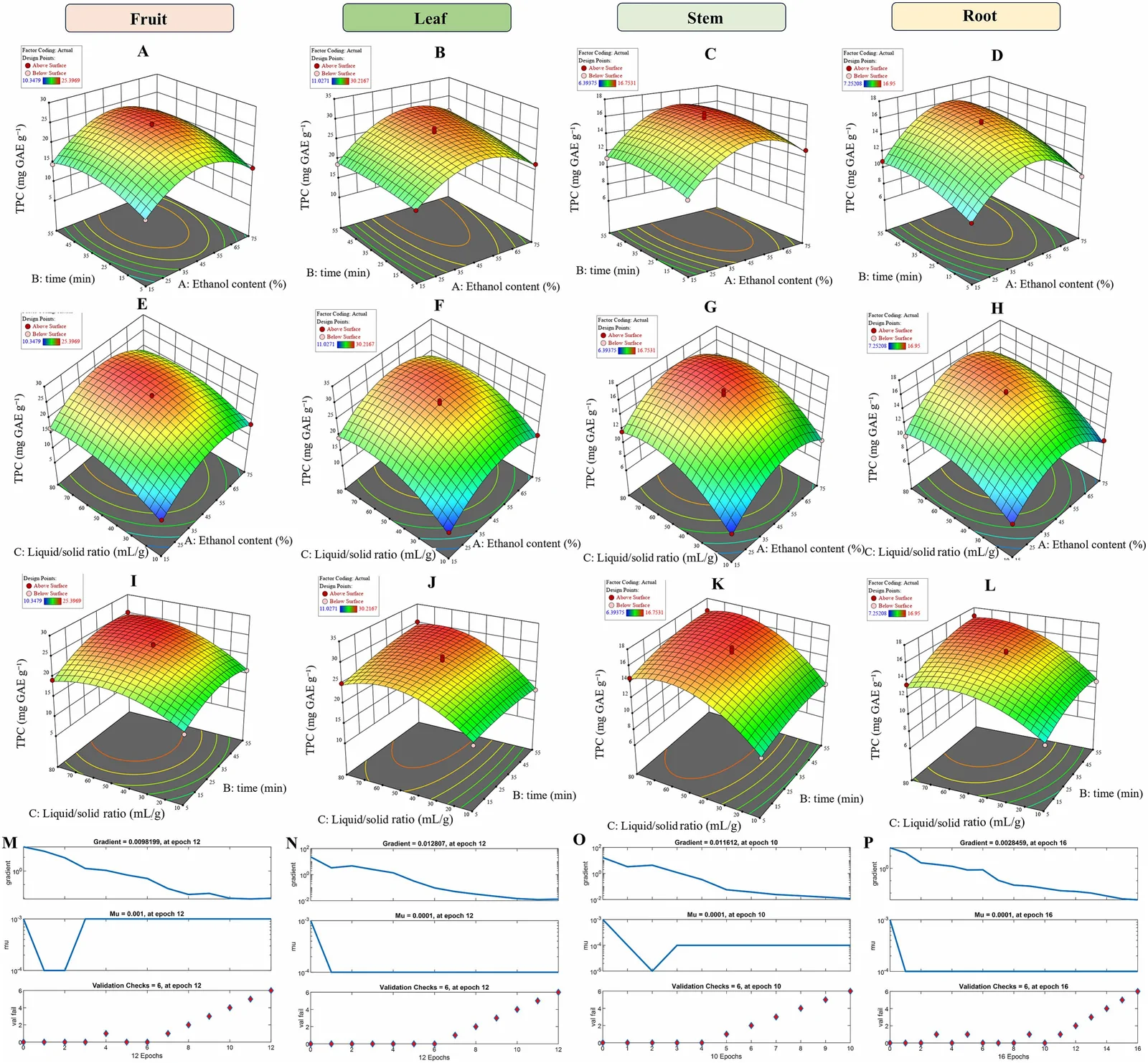
Three-dimensional RSM plots and ANN training diagnostic curves for UAE parameter optimization of TPC from four *A. manihot* bio-wastes. (A-D, E-H, I-L) RSM surfaces for fruit, leaf, stem and root bio-wastes, showing interactive effects of ethanol content and ultrasound time, ethanol content and LSR, ultrasound time and LSR, respectively. (M−P) ANN training curves for fruit, leaf, stem and root, including gradient evolution, Mu and validation check failure plots to evaluate model convergence and stability.

To further capture the complex nonlinear relationships between extraction parameters and responses, BP-ANN models were constructed for each tissue [6]. The training diagnostic curves (Fig. 7**M–P;** Supplementary Figs. 13–16) demonstrated stable convergence, with overall R values ranging from 0.90388 to 0.94358. A comparative error analysis (Supplementary Table 9) indicated that the BP-ANN models yielded lower internal prediction errors compared to the quadratic regression models. For example, the MSE for fruit TPC decreased from 0.5220 (RSM) to 0.0125 (BP-ANN). However, it must be explicitly noted that the test set for the BP-ANN was drawn from the same experimental design space (i.e., internal hold-out data), rather than from truly independent external datasets. Therefore, while the BP-ANN exhibits superior capability in interpolating highly nonlinear relationships within the given design space, its extrapolation performance remains unverified. To mitigate overfitting, an early stopping criterion was employed during training. Given that ANN is primarily a flexible black-box fitting tool, its higher internal predictive accuracy does not invalidate the practical value of RSM. The BP-ANN's advantage stems from its flexible, data-driven architecture without pre-specified functional forms [6], [8]. In contrast, the quadratic models, though inherently limited in capturing highly complex nonlinear relationships [33], provide explicit mathematical equations. These equations clearly reveal the main effects, interaction effects, and optimal conditions, which are essential for guiding actual production and process control. Collectively, the RSM serves as the primary methodological framework for optimization and mechanistic interpretation, while the BP-ANN merely serves as a complementary tool to confirm the presence of nonlinear patterns, with both approaches ultimately indicating the same optimal extraction regions.

### 3.6 Characterization of polyphenol extracts from different *a. Manihot* bio-wastes

#### 3.6.1 UV–Vis spectral analysis

The macroscopic morphology and ultraviolet absorption characteristics of fruit, leaf, stem and root polyphenol extracts were firstly characterized (Fig. 8**A and 8B**). At a uniform concentration of 0.5 mg/mL, obvious discrepancies in solution color were observed among four extracts, leaf extract exhibited the deepest yellow–brown color, while root extract presented the lightest pale yellow hue, preliminarily reflecting the divergence in total polyphenol content and constituent composition across different tissues. As shown in the UV–Vis spectra (Fig. 8**B**), all four samples displayed strong continuous absorption within the 200 – 400 nm ultraviolet region, which is the characteristic absorption band of phenolic hydroxyl and aromatic ring structures in polyphenol molecules [7], [62]. The maximum absorption peaks mainly concentrated below 300 nm, consistent with the typical UV absorption feature of flavonoid and phenolic acid derivatives [7], [62]. Meanwhile, leaf extract possessed higher absorbance values throughout the full scanning range, which matched the foregoing quantitative detection result that leaf had the highest TPC yield, whereas root extract showed the minimum absorbance, corresponding to its lowest polyphenol content. Such spectral differences further verified the compositional divergence of polyphenolic ingredients derived from four *A. manihot* bio-wastes.

**Fig. 8:**
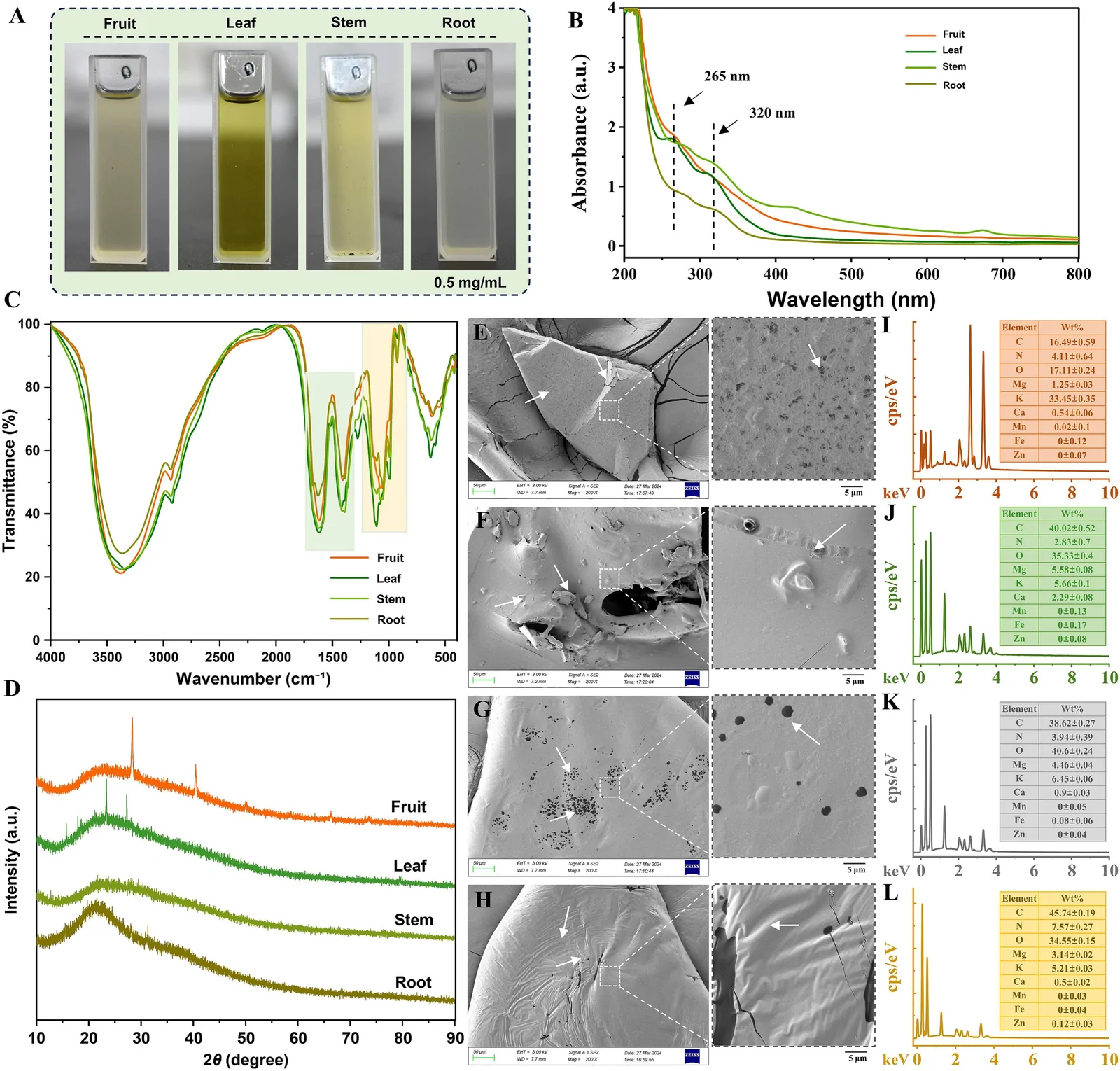
Characterization of extracts from different bio-wastes of *A. manihot*. (A) Visual appearance of the extracts at a concentration of 0.5 mg/mL under daylight. (B) UV–Vis absorption spectra of the extracts. (C) FTIR spectra of the extracts. (D) XRD patterns of the extracts. (E–H) SEM micrographs of the extracts from fruit (E), leaf (F), stem (G), and root (H). (I–L) EDS elemental analysis of the corresponding extracts.

#### 3.6.2 FTIR spectroscopic analysis

FTIR spectroscopy was adopted to identify characteristic functional groups of polyphenolic extracts from four raw materials (Fig. 8**C**). All samples shared analogous overall infrared absorption profiles with several identical characteristic absorption bands, proving that the dominant chemical skeleton of polyphenols from fruit, leaf, stem and root was consistent [63], [64]. The broad strong absorption near 3200 – 3600 cm^−1^ originated from stretching vibration of phenolic –OH groups, which was the most typical characteristic peak of polyphenol substances, multiple moderate absorption signals at 2800–3000 cm^−1^ were ascribed to C–H stretching vibration of aliphatic hydrocarbon on aromatic rings [63], [64]. Distinct absorption peaks around 1610 – 1650 cm^−1^ corresponded to C=C and C=O stretching vibration of benzene ring and flavonoid carbonyl structure, and successive absorption bands ranging from 1000 to 1500 cm^−1^ were assigned to C–O stretching vibration of phenolic hydroxyl and ether bonds [63], [64], [65], [66]. Minor peak shifts and absorbance intensity differences among four curves implied subtle distinctions in the substitution mode of phenolic hydroxyl, polymerization degree of polyphenols and auxiliary impurity composition between different parts, which arose from the disparate metabolic accumulation of secondary metabolites in various plant tissues [6], [7], [63], [65].

#### 3.6.3 XRD analysis

Crystalline properties of four polyphenol extracts were characterized *via* X-ray diffraction (Fig. 8**D**). All extracts displayed one sharp diffraction peak near 2*θ* = 20–30° together with broad amorphous diffuse scattering backgrounds, demonstrating that the polyphenol extracts existed in a mixed state of partial microcrystalline structure and dominant amorphous form [7]. Fruit extract presented the highest diffraction peak intensity at the characteristic position, indicating relatively higher crystallinity of its polyphenol constituents [7], [67], leaf, stem and root extracts showed gradually weakened peak height sequentially, accompanied by enhanced amorphous diffuse halo, which was attributed to varied polyphenol polymerization degree and different contents of soluble saccharide, protein and other amorphous impurities introduced during extraction [68], [69]. The diversified crystallinity features reflected the difference in molecular aggregation state of polyphenols separated from four *A. manihot* bio-wastes.

#### 3.6.4 SEM and EDS elemental analysis

Surface micromorphology and elemental composition of four polyphenol extracts were characterized *via* SEM-EDS elemental mapping (Fig. 8**E–L**). Although the overall macroscopic morphology of the four extracts showed no prominent divergence at low magnification, obvious fine structural distinctions were observed at microscale. The fruit-derived extract (Fig. 8**E**) formed stacked massive aggregates scattered with sporadic surface fissures, leaf extract (Fig. 8**F**) consisted of irregular folded lamellar fragments with scattered micropores, stem extract (Fig. 8**G**) existed as loosely agglomerated fine particles mixed with tiny sporadic granular impurities, root extract (Fig. 8**H**) presented relatively intact continuous film-like texture decorated with sparse shallow surface wrinkles. Such subtle microstructural disparities stem from inherent differences in the polarity, polymerization degree of intrinsic polyphenols and coexisting minor impurities across diverse plant tissues [6], [7]. Corresponding EDS elemental mapping (Fig. 8**I–L**) indicated carbon and oxygen dominated the elemental composition of all specimens, consistent with the molecular structural features of polyphenols built by aromatic carbon skeletons and abundant oxygen-bearing functional groups [7], [70]. Minor mineral elements including Mg, Ca, Mn, Fe and Zn were universally detectable across all four extracts, originating from inherent mineral nutrition of raw plant materials and trace inorganic residues generated during UAE processing [71]. Variations in the mass proportion of these trace inorganic elements further differentiate the chemical compositional features of polyphenol extracts from *A. manihot* fruit, leaf, stem and root.

### 3.7 *In vitro* antioxidant activity of polyphenol extracts

Four classic *in vitro* assays (DPPH, ABTS, FRAP, and hydroxyl radical scavenging) were used to evaluate polyphenol extracts from fruit, leaf, stem, and root wastes of *A. manihot*. Ascorbic acid and Trolox served as positive controls. Concentration-response curves and quantitative indices (IC_50_ for radical scavenging, IC_1.0_ for FRAP) are compiled in Fig. 9**A–H**. All extracts showed typical dose-dependent responses, radical scavenging rates and FRAP absorbance increased with concentration before plateauing.

**Fig. 9:**
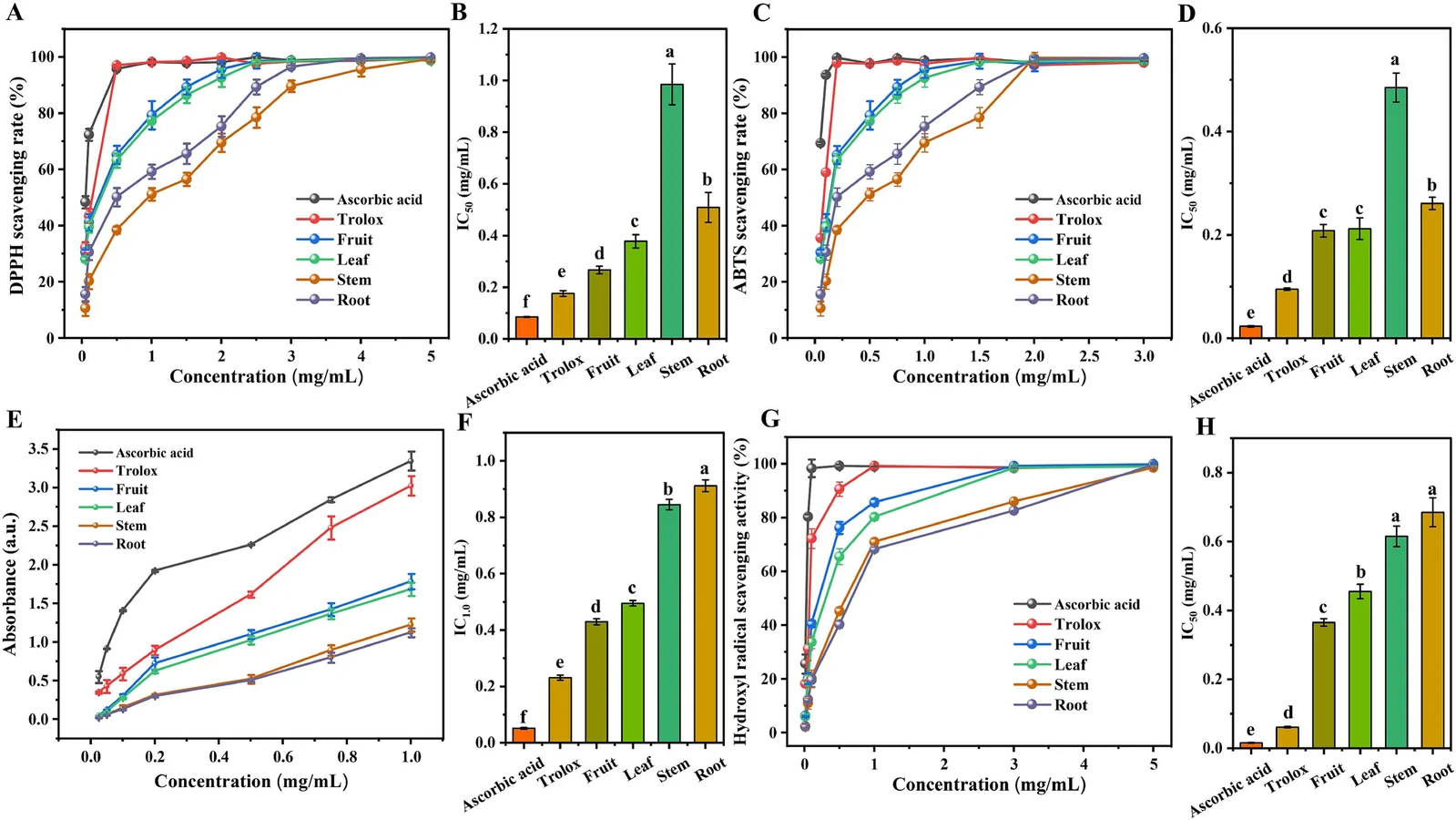
*In vitro* antioxidant activities of extracts from different bio-wastes of *A. manihot*. (A) DPPH radical scavenging activity and (B) corresponding IC_50_ values. (C) ABTS radical scavenging activity and (D) corresponding IC_50_ values. (E) FRAP activity and (F) corresponding FRAP values (IC_1.0_, concentration required to achieve an absorbance of 1.0 a.u.). (G) Hydroxyl radical scavenging activity and (H) corresponding IC_50_ values. Data are presented as mean ± standard deviation (n = 3). Different lowercase letters above the bars indicate significant differences (*p* < 0.05).

In DPPH assays (Fig. 9**A, and 9B**), ascorbic acid had the lowest IC_50_ among all samples, and Trolox showed stronger activity than all plant extracts. Among botanical samples, fruit extract had the lowest IC_50_, significantly superior to leaf, root and stem (*p* < 0.05). Specifically, the IC_50_ of fruit extract was approximately 2.5-fold lower than that of stem extract. Leaf and root extracts exhibited intermediate activity. ABTS results (Fig. 9**C and 9D**) showed the same ranking, fruit and leaf extracts again ranked first among the four tissues, reflecting their strong electron-donating capacity. This superior antioxidant property correlates with the highest total phenolic content of leaf and fruit extracts from optimized extraction, as abundant phenolic hydroxyl groups efficiently stabilize ABTS cation radicals [72]. FRAP assay (Fig. 9**E and 9F**) measured ferric reducing ability *via* IC_1.0_. Ascorbic acid gave the lowest IC_1.0_, followed by Trolox. Among plant extracts, fruit and leaf displayed markedly lower IC_1.0_ values than stem and root (*p* < 0.05), and their reducing power was close to that of Trolox. Notably, fruit extract achieved the lowest IC_1.0_, followed by leaf extract. Although the total phenolic content of fruit (16.72 mg/g) was lower than that of leaf (28.93 mg/g), its FRAP activity was superior. This indicates that lignified fruit tissues are rich in condensed tannins and specific phenolic derivatives such as proanthocyanidins [6], [73]. These components have strong electron-donating ability and exert prominent ferric reducing effects, which are not positively correlated with the TPC of the extracts [6], [73]. Consequently, stem extract may be particularly valuable for applications requiring ferric reduction rather than radical scavenging. In contrast, root extract had the highest IC_1.0_, indicating the weakest reducing capacity, likely due to its lower polyphenol diversity and higher proportion of bound phenolics [20]. Hydroxyl radical scavenging (Fig. 9**G and 9H**) is critical for in vivo antioxidant potential. Ascorbic acid and Trolox again outperformed all plant extracts. Among botanical samples, fruit retained the lowest IC_50_, approximately 1.8-fold lower than that of root extract, with stem and leaf extracts ranking in between. Significant intergroup differences (*p* < 0.05) confirmed tissue-specific antioxidant activity due to differential polyphenol accumulation and composition.

Overall, the antioxidant potency matching the TPC gradient from extraction optimization. This discrepancy highlights that different antioxidant mechanisms (hydrogen atom transfer *vs.* electron transfer) are governed by distinct polyphenol structural features [6], [74], [75]. Variations in cell wall structure, lignification degree, and secondary metabolic pathways among tissues lead to distinct polyphenol polymerization patterns, hydroxyl substitution modes, and impurity profiles, ultimately diversifying antioxidant performance [6], [74], [75]. All four waste-derived extracts possess favorable *in vitro* antioxidant activity, especially fruit and leaf polyphenols, which can serve as cost-effective natural alternatives for food and cosmetic industries, facilitating full cyclic utilization of agricultural by-products.

### 3.8 UHPLC-MS/MS-Based polyphenolic metabolite profiling

To systematically elucidate the intrinsic chemical basis for the divergent antioxidant performance of polyphenol extracts from fruit, leaf, stem, and root bio-wastes of *A. manihot*, non-targeted UHPLC-MS/MS metabolomics was employed to comprehensively profile the constituent polyphenolic metabolites across the four tissues. Multivariate statistical analyses, including PCA, PLS-DA, VIP screening, hierarchical clustering, and differential metabolite screening, were further performed to clarify tissue-specific accumulation characteristics of polyphenolic ingredients (Fig. 11**,** Supplementary Figs. 17–21).

UHPLC chromatograms acquired under positive and negative electrospray ionization modes intuitively revealed prominent disparities in peak distribution, retention time and peak intensity among four sample groups (Fig. 10**A and 10B**). A large batch of characteristic chromatographic peaks corresponding to flavonoids, phenolic acids, tannins and other phenolic derivatives were annotated according to standard molecular weight, fragmentation pathway and published phytochemical data. Representative marker substances covering rutin, quercetin, morin, kaempferol, gallic acid, chlorogenic acid derivatives, ferulic acid and ellagic acid were successfully localized on chromatogram curves, which verified that polyphenolic constituents dominated the chemical composition of all four extracts, consistent with the foregoing FTIR functional group characterization results. Nevertheless, obvious divergence in peak abundance of identical characteristic compounds could be observed, preliminarily demonstrating distinct metabolic accumulation preference across different bio-wastes of *A. manihot*.

**Fig. 10:**
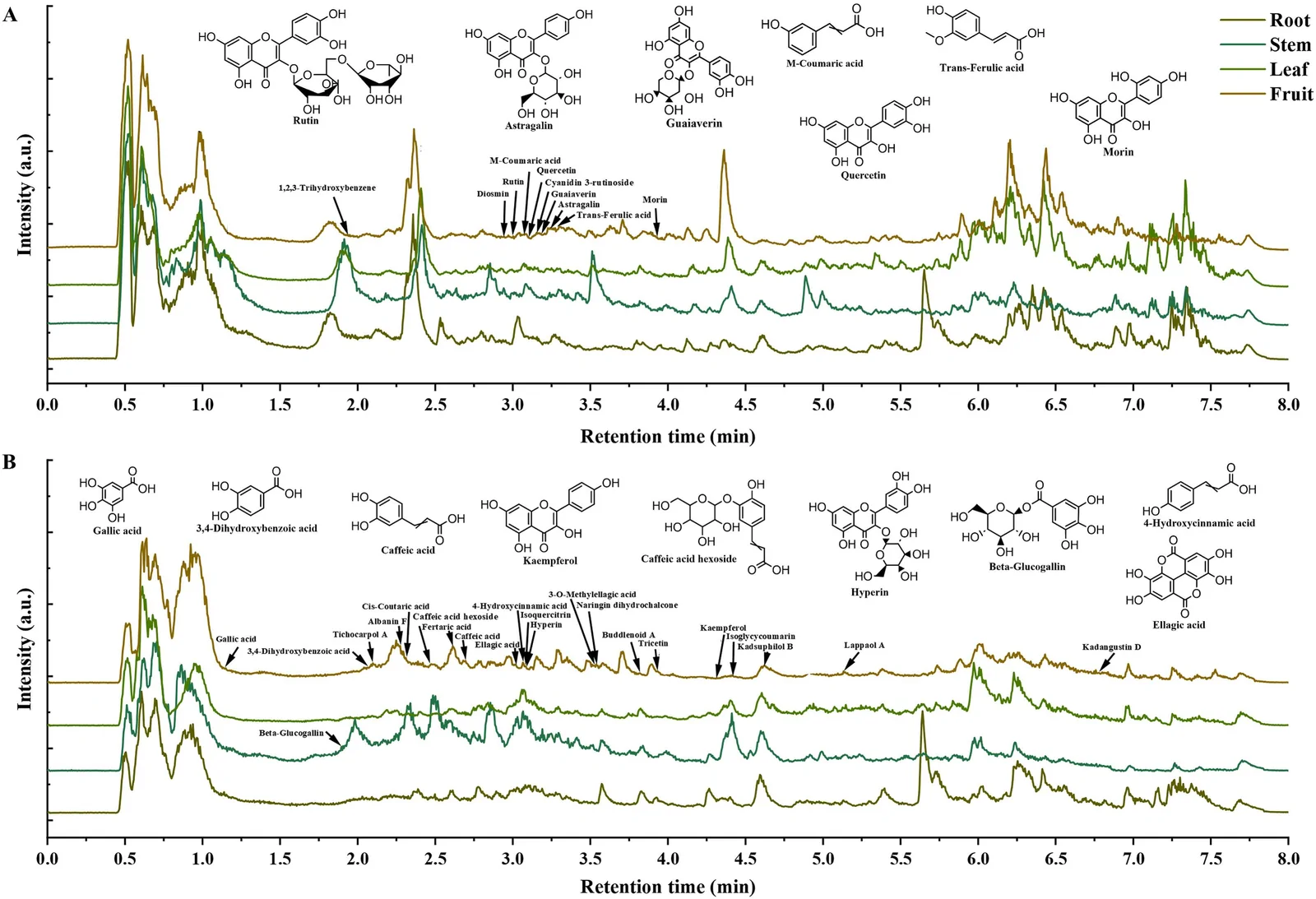
UHPLC chromatograms of extracts from different bio-wastes of *A. manihot*. (A) Positive ion mode chromatograms. (B) Negative ion mode chromatograms. The identified major polyphenolic compounds are labeled with their corresponding chemical structures.

Global secondary metabolite classification statistics (Fig. 11**A–B**) indicated that a total of 508 annotated secondary metabolites were distributed across multiple chemical subclasses. Flavonoids (298 species, accounting for 57.75 %) and phenolic acid derivatives (200 species, 38.76 %) occupied the absolute dominant proportion, while tannins, terpenoids, coumarins, alkaloids, and lignins constituted only minor components. As listed in Table 2**, 31** high-abundance and representative polyphenolic metabolites were further qualitatively identified based on accurate *m*/*z* values, molecular formulas, and CAS database matching. To ensure rigorous annotation confidence, their Metabolomics Standards Initiative (MSI) levels were strictly assigned (Supplementary Table 10). Specifically, 26 key markers were unambiguously validated as MSI Level 1 using authentic standards (matching both retention times and MS^2^ fragments), while the remaining 5 were assigned as MSI Level 2 based on high-confidence database matching without standard verification. The annotated core compounds primarily consisted of flavonoid glycosides, flavonols, cinnamic acid derivatives, tannins, and benzoic acid derivatives, with rutin, quercetin, kaempferol, diosmin, ellagic acid, and gallic acid identified as the most abundant polyphenolic constituents. These well-documented antioxidant polyphenols provide direct chemical evidence for the strong free radical scavenging ability and reducing capacity of the four *A. manihot* bio-waste extracts [7], [8], [10]. The detailed experimental MS/MS fragmentation spectra and database matching mirror plots for these core markers are provided in Supplementary Figs. 17 and 18, respectively, confirming the high reliability of our annotations. Due to the limited commercial availability of authentic standards for all derivatives, quantification was performed via relative peak area normalization rather than absolute quantification, and all QC sample RSD values were well below 30 % (Supplementary Table 10). Such compositional distribution directly explains the robust free radical scavenging and reducing activity detected in the preceding *in vitro* antioxidant assays, as flavonoid and phenolic acid skeletons are endowed with abundant phenolic hydroxyl groups responsible for antioxidant bioactivity [7], [8], [10]. Principal component analysis (PCA, Fig. 11**C**) displayed satisfactory sample clustering and clear group separation: the four tissue-derived samples assembled into independent clusters without cross-overlapping, with PC1 and PC2 collectively explaining over 60 % of total metabolic variation, statistically confirming that the metabolite fingerprint could reliably distinguish fruit, leaf, stem, and root raw materials. The supervised PLS-DA model further enhanced group discrimination (Fig. 11**D**), with obvious spatial separation of the four groups on component coordinates and QC samples tightly clustered near the central region, proving a stable detection system and high reproducibility of metabolomics quantification data. Variable importance in projection (VIP) screening (Fig. 11**F**) identified a series of core differential polyphenolic markers with VIP > 1; these high-contribution compounds—including kaempferol glycosides, hyperin, rutin, ellagic acid, and caffeic acid derivatives—acted as the primary chemical markers to differentiate the four tissue extracts. Venn diagram analysis (Fig. 11**E**) visualized shared and unique differential metabolites among pairwise comparison groups. A total of 39 core common differential metabolites existed across all six comparison sets, while dozens of tissue-specific enriched metabolites were separately detected in leaf, fruit, stem, and root samples. Hierarchical clustering heatmaps of the overall polyphenolic metabolites (Supplementary Fig. 19), top-abundant flavonoids (Supplementary Fig. 20**A**), phenolic acids (Supplementary Fig. 20**B**), and tannins (Supplementary Fig. 20**C**) further quantified the abundance variation of dominant polyphenols. Leaf samples presented widespread high accumulation of most flavonol glycosides, hydroxycinnamic acid, and gallotannin derivatives, reflected by continuous red blocks on the heatmaps. Fruit extracts featured specific enrichment of morin, *trans*-ferulic acid, and related derivatives. Stem tissue was characterized by a high relative content of condensed tannins and simple benzoic acid derivatives. Root extracts accumulated abundant small-molecule phenolic acids such as *p*-hydroxyphenylacetic acid, alongside relatively low flavonoid abundance, accompanied by an overall blue-dominated low-abundance distribution for most flavonoid aglycones and glycosides. These tissue-specific enrichment rules matched the gradient ranking of *in vitro* antioxidant capacity determined in Section 3.7, clarifying that the superior antioxidant potency of fruit and leaf extracts originated from the highest total diversity and accumulation level of flavonoid and phenolic acid metabolites.

**Fig. 11:**
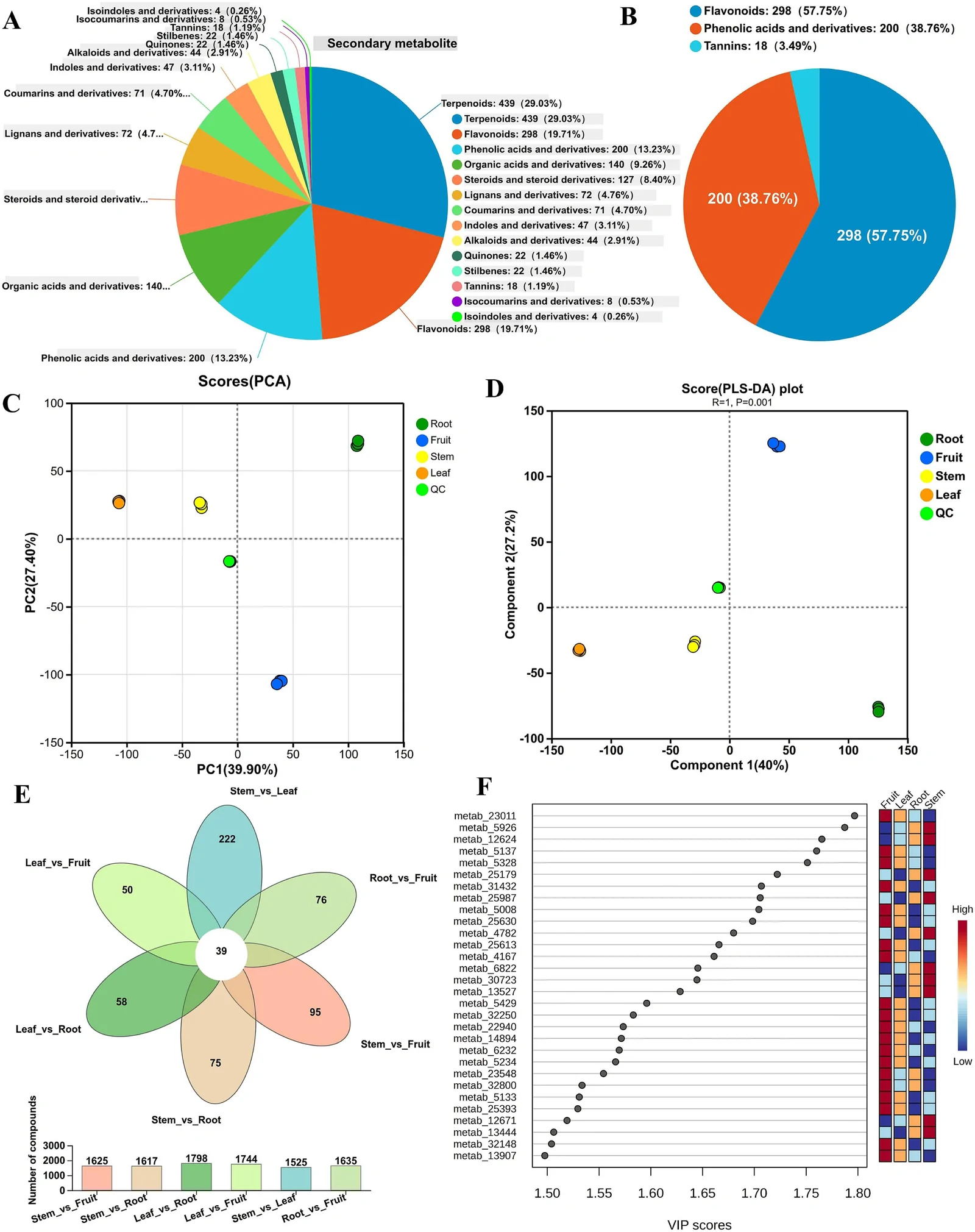
Comprehensive metabolomics analysis of extracts from different bio-wastes of *A. manihot*. (A) Classification of all identified secondary metabolites, (B) classification of phenolic compounds, (C) PCA score plot of metabolites, (D) PLS-DA score plot of metabolites, (E) Venn diagram of differentially expressed metabolites, and (F) VIP analysis of polyphenolic metabolites.

**Table 2:** Representative high-abundance polyphenolic compounds identified in optimized ultrasound-assisted extracts of *A. manihot* bio-waste *via* UHPLC-MS/MS analysis.

ID	Compounds	Formula	*m*/*z*	CAS Number	Class
1	Diosmin	C_28_H_32_O_15_	609.181	520-27-4	Flavonoid glycosides
2	Quercetin	C_15_H_10_O_7_	303.050	117-39-5	Flavonols
3	Guaiaverin	C_20_H_18_O_11_	435.092	22255-13-6	Flavonoid glycosides
4	Morin	C_15_H_10_O_7_	303.050	480-16-0	Flavonols
5	*Trans*-Ferulic acid	C_10_H_10_O_4_	177.054	1135-24-6	Cinnamic acids and derivatives
6	Astragalin	C_21_H_20_O_11_	449.107	480-10-4	Flavonoid glycosides
7	Cyanidin 3-rutinoside	C_27_H_31_O_15_	595.165	28338-59-2	Anthocyanins
8	*M*−Coumaric acid	C_9_H_8_O_3_	165.054	14755-02-3	Cinnamic acids and derivatives
9	Rutin	C_27_H_30_O_16_	611.160	153-18-4	Flavonoid glycosides
10	Narcissin	C_28_H_32_O_16_	625.176	604-80-8	Flavonoid glycosides
11	1,2,3-Trihydroxybenzene	C_6_H_6_O_3_	127.039	87-66-1	Phloroglucinols and derivatives
12	Caffeic acid hexoside	C_15_H_18_O_9_	341.088	14364-08-0	Cinnamic acids and derivatives
13	Fertaric acid	C_14_H_14_O_9_	385.077	74282-22-7	Cinnamic acids and derivatives
14	Caffeic acid	C_9_H_8_O_4_	179.035	501-16-6	Cinnamic acids and derivatives
15	Ellagic acid	C_14_H_6_O_8_	300.999	476-66-4	Tannins
16	4-Hydroxycinnamic acid	C_9_H_8_O_3_	163.040	501-98-4	Cinnamic acids and derivatives
17	Isoquercitrin	C_21_H_20_O_12_	463.088	482-35-9	Flavonoid glycosides
18	Kaempferol	C_15_H_10_O_6_	285.040	520-18-3	Flavonols
19	Lappaol A	C_30_H_32_O_9_	535.197	62333-08-8	Benzofuran flavonoids
20	Kadangustin D	C_34_H_38_O_10_	605.240	1013633-22-1	Tannins
21	Kadsuphilol B	C_31_H_32_O_10_	563.192	947256-77-1	Tannins
22	Tricetin	C_15_H_10_O_7_	301.035	520-31-0	Flavones
23	Buddlenoid A	C_30_H_26_O_13_	593.130	142750-32-1	Flavonoid glycosides
24	Naringin dihydrochalcone	C_27_H_34_O_14_	641.209	18916-17-1	Flavonoid glycosides
25	3-O-Methylellagic acid	C_15_H_8_O_8_	315.014	51768-38-8	Tannins
26	*Cis*-Coutaric acid	C_13_H_12_O_8_	355.067	67920-37-0	Cinnamic acids and derivatives
27	Tichocarpol A	C_9_H_10_O_7_S	261.007	735328-83-3	Phenylpropanoic acids and derivatives
28	3,4-Dihydroxybenzoic acid	C_7_H_6_O_4_	153.019	99-50-3	Benzoic acids and derivatives
29	*Beta*-Glucogallin	C_13_H_16_O_10_	331.067	554-37-0	Tannins
30	Gallic acid	C_7_H_6_O_5_	169.014	149-91-7	Benzoic acids and derivatives
31	Hyperin	C_21_H_20_O_12_	927.182	482-36-0	Flavonoid glycosides

Note: Selected high‑abundance polyphenols (flavonoids, phenolic acids, tannins, and related derivatives) were annotated by *m*/*z*, molecular formula, CAS number, and database matching.

Six groups of pairwise comparative volcano plots (Supplementary Fig. 21**A–F**) systematically quantified upregulated and downregulated differential polyphenols between any two tissues, with significantly regulated metabolites defined as |log_2_FC| > 2 and *p* < 0.05. In the comparison of Fruit *vs* Leaf, large quantities of flavonoid glycosides were markedly upregulated in the leaf group and downregulated in fruit. The Root *vs* Fruit comparison revealed that most medium-polarity flavonoids were significantly depleted in root tissues, whereas several short-chain phenolic acids were distinctly enriched in root samples. The Stem *vs* Leaf contrast demonstrated that leaf possessed massively upregulated hydroxycinnamic acid and flavonol derivatives, while stem selectively accumulated polymeric tannin ingredients. Collectively, leaf exhibited the largest number of significantly upregulated antioxidant-related polyphenolic metabolites across all pairwise comparisons, root owned the maximum quantity of downregulated flavonoid compounds, and fruit and stem presented intermediate differential regulation characteristics.

### 3.9 Mantel correlation analysis between polyphenol constituents and *in vitro* antioxidant capacity

To evaluate the overall association between polyphenol classes and antioxidant activities, the Mantel test was selected. Unlike predictive modeling approaches that focus on feature ranking or explanatory power, the Mantel test directly quantifies the correlation between two distance-based similarity matrices, making it particularly suitable for high-dimensional metabolomic data in which variables are highly collinear and the number of features exceeds the number of samples [76], [77]. In this study, distance matrices were constructed using Euclidean distances based on standardized polyphenol abundance and antioxidant activity data, and significance was assessed with 999 permutations. To uncover the intrinsic ingredient–activity linkage between characteristic polyphenols and four antioxidant indicators (DPPH, ABTS, FRAP, hydroxyl radical scavenging capacity), Mantel test coupled with Pearson correlation analysis was conducted using quantitative metabolomic and antioxidant data (Fig. 12). High-abundance polyphenols were grouped into phenolic acids, flavonoids and tannins for grouped correlation calculation to identify which phenolic classes were most strongly correlated with the antioxidant efficacy of *A. manihot* bio-waste extracts. As shown in Fig. 12**A**, nearly all major phenolic acid derivatives correlated positively and significantly with all four antioxidant assays. Representative molecules such as gallic acid, caffeic acid, ferulic acid, 3,4-dihydroxybenzoic acid and dihydrocaffeic acid exhibited thick linkage lines and warm-colored positive-correlation blocks. The phenolic hydroxyl groups on their aromatic rings readily donate hydrogen to terminate radical chain reactions, enabling potent radical scavenging and ferric reduction, a few individual phenolic acids showed trivial non-significant correlations owing to their low endogenous accumulation in specific tissues [76], [77]. Flavonoids presented the broadest and strongest positive correlations against all antioxidant metrics (Fig. 12**B**). Key flavonol glycosides (rutin, isoquercitrin, astragalin, hyperin), quercetin, morin and kaempferol derivatives were markedly positively associated with four testing indices. Rich conjugated double bonds and polyhydroxy substitutions on flavonoid skeletons confer superior electron-donating capacity, rationalizing the top-tier antioxidant performance of flavonoid-rich leaf extracts observed earlier and suggesting that flavonoids were the most strongly correlative fraction associated with the extract antioxidant potency [76], [77]. For tannins (Fig. 12**C**), ellagic acid, beta-glucogallin and oligomeric tannins also exerted significant positive correlations with antioxidant activity, yet their overall correlation magnitude was inferior to flavonoids and phenolic acids. Multi-hydroxyl cyclic structures of polymeric tannins synergistically facilitate radical neutralization and ferric reduction, whereas partial high-molecular tannins yielded low correlation coefficients because steric hindrance restricts their binding with radical substrates [20], [78].

**Fig. 12:**
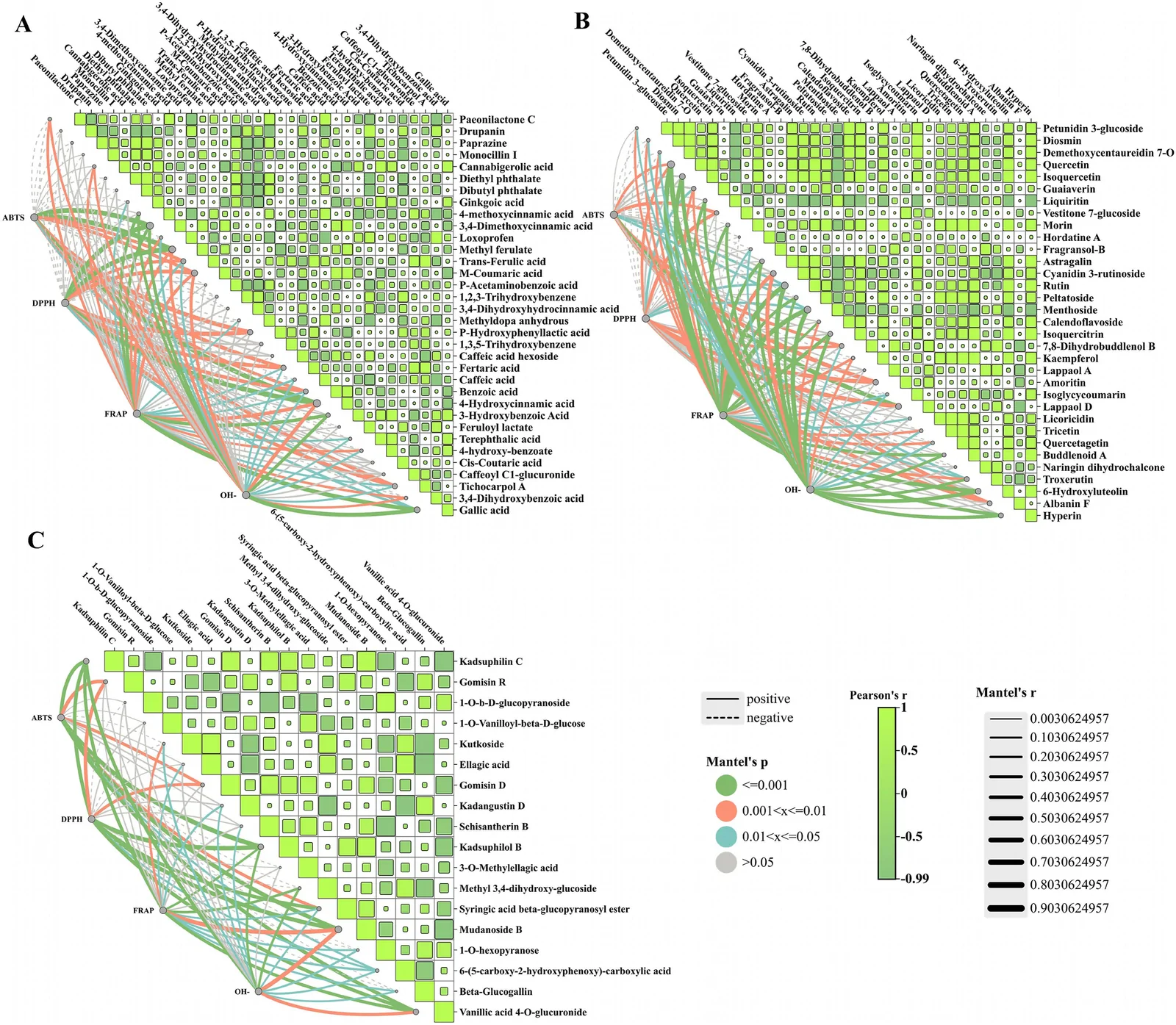
Mantel test analysis showing the correlations between major polyphenolic compounds (top 100 in abundance) and antioxidant activities of extracts from different bio-wastes of *A. manihot*. (A) Correlations between phenolic acids and antioxidant activities. (B) Correlations between flavonoids and antioxidant activities. (C) Correlations between tannins and antioxidant activities. The color gradient denotes Pearson’s correlation coefficients. Solid lines indicate positive correlations and dashed lines indicate negative correlations; line color and width represent Mantel’s p and Mantel’s r, respectively.

Collectively, flavonoids followed by phenolic acids exhibited the strongest correlative associations with the antioxidant property of *A. manihot* polyphenol bio-waste extracts, while tannins played an auxiliary correlative role. It should be noted that the Mantel test reveals correlative associations rather than definitive causal relationships or exact contribution weights. The Mantel correlation outcomes corroborate the foregoing ranking rule of antioxidant capacity and metabolomics differential accumulation characteristics, further illuminating the chemical essence of tissue-dependent antioxidant divergence.

### 3.10 Comprehensive greenness assessment and industrial relevance

To quantitatively validate the “green recovery” claim and assess practical industrial relevance, the environmental impacts of the six extraction systems were evaluated using ComplexGAPI (Fig. 13**A**) and AGREEprep (Fig. 13**B**). In the ComplexGAPI assessment (Fig. 13**A**), the ultrasound-assisted water/ethanol binary solvent (UA-E/W BS) and ultrasound-assisted water (UA-W MS) achieved the highest scores (66). The AGREEprep assessment (Fig. 13**B**) further corroborated this, where UA-E/W BS scored the highest (0.63), notably outperforming its non-ultrasonic control (NUA-E MS, 0.54). This advantage is primarily attributed to the ultrasound-induced rapid extraction, which reduces the specific energy consumption to approximately 0.005 kWh per sample, thereby minimizing the life-cycle carbon footprint. Regarding solvent recovery and cost estimation, both water/ethanol and emerging DES align with green chemistry principles, but they present distinct operational characteristics. While DES systems offer highly tunable properties and low volatility, their inherently high viscosity can limit mass transfer during extraction and complicate downstream product separation. In contrast, the water/ethanol system utilized here offers low viscosity for facile mass transfer, minimal material costs, and operational simplicity, as ethanol can be readily recovered via conventional rotary evaporation. Furthermore, the generated aqueous waste (approx. 30 mL per batch) is non-toxic, and the solid plant residues can be repurposed for bioenergy, supporting a zero-waste biorefinery model. For industrial scale-up, conventional ultrasonic baths present limitations regarding the uniformity of acoustic energy density distribution (e.g., standing wave attenuation and dead zones at larger volumes); however, this scale-up challenge can be mitigated by transitioning from bath-type systems to continuous-flow or probe-type ultrasonic reactors. Overall, the combined results confirm that the water/ethanol UAE system offers a practically viable, easily recoverable, and environmentally sustainable strategy for the valorization of *A. manihot* bio-wastes.

**Fig. 13:**
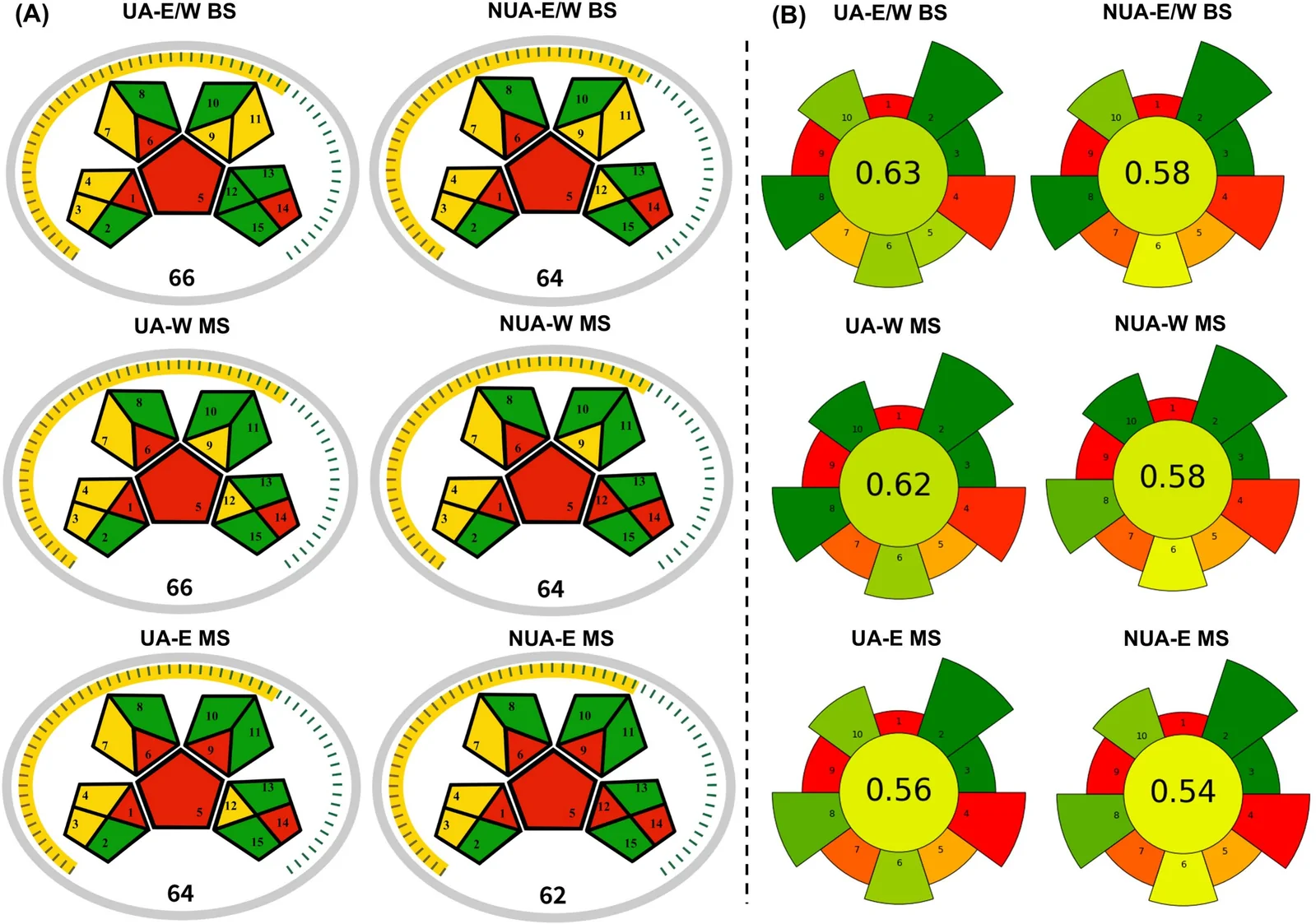
Comprehensive greenness assessment of different extraction systems using ComplexGAPI and AGREEprep metrics. (A) ComplexGAPI pictograms and total scores for the six extraction conditions. (B) AGREEprep pictograms and overall scores for the corresponding conditions.

## 4 Conclusion

In this study, a green UAE process was developed for polyphenol recovery from four *A. manihot* bio-waste tissues (fruit, leaf, stem, and root). The novelty of this work lies in the systematic comparison of four tissues with different lignification degrees, combined with energy-calibrated sonication, kinetic modeling, SEM, and metabolomics. Calorimetric calibration established a linear acoustic energy output (R^2^ = 0.9942), and a medium acoustic energy density of 113.6 W/L was selected, ensuring stable cavitation. KI dosimetry quantified cavitation activity, with triiodide accumulation fitted by both linear and nonlinear models. Water/ethanol binary solvent with UAE gave the highest polyphenol yields and antioxidant activities across all tissues, with a net increment of 3.87 mg GAE/g for leaf. Pseudo-second-order kinetics best described extraction, and UAE increased rate constants, indicating reduced mass transfer resistance. SEM revealed ultrasound-induced cell wall disruption, more pronounced in thin-walled leaf and fruit than in lignified stem and root, which correlated with extraction efficiency. Extraction parameters were optimized by RSM and BP-ANN, with BP-ANN showing superior predictive accuracy. Antioxidant assays ranked fruit > leaf > stem > root. UHPLC-MS/MS metabolomics identified 508 polyphenols dominated by flavonoids (57.75 %) and phenolic acids (38.76 %), with tissue-specific accumulation. Mantel test revealed that flavonoids and phenolic acids were strongly correlated with antioxidant activity, with tannins playing auxiliary roles. Furthermore, quantitative greenness assessments confirmed the ultra-low energy consumption and high sustainability of the extraction process. This integrated acoustic, kinetic, microstructural, and metabolomic evidence provides a mechanistic basis for ultrasound-enhanced polyphenol recovery, and demonstrates the potential of UAE for green recovery of polyphenols from agricultural bio-wastes. The systematic comparison across tissues with different lignification degrees offers new insights for differentiated utilization of plant bio-wastes. However, this study is currently limited to laboratory-scale experiments, and a comprehensive techno-economic assessment and life-cycle assessment were not performed. Future research should focus on scaling up the process using continuous-flow ultrasonic reactors, evaluating industrial solvent recovery efficiency, and validating the in vivo bioactivity of the recovered polyphenols.

## CRediT authorship contribution statement

**Wen-zhang Qian:** Writing – review & editing, Writing – original draft, Visualization, Validation, Supervision, Software, Resources, Methodology, Formal analysis, Data curation, Conceptualization. **Li Zhao:** Supervision, Software, Resources, Methodology, Investigation, Funding acquisition, Formal analysis, Data curation, Conceptualization. **Jin-you Yao:** Validation, Software, Resources. **Pei-wen Liang:** Software, Resources, Methodology. **Yu-han Hu:** Software, Resources. **Yi-xiao Xiao:** Visualization, Validation, Supervision, Software, Resources, Methodology, Investigation. **Shun Gao:** Writing – original draft, Validation, Supervision, Software, Resources, Project administration, Methodology, Investigation, Funding acquisition, Formal analysis, Conceptualization.

## Declaration of competing interest

The authors declare that they have no known competing financial interests or personal relationships that could have appeared to influence the work reported in this paper.

## Appendix A Supplementary data

Supplementary Data 1 Supplementary data associated with this article, including additional figures (Supplementary Figs. 1–21) and tables (Supplementary Tables 1–10).
