# Supplementary material for: Ultrasound‑enhanced green recovery and antioxidant potential of polyphenols from four Abelmoschus manihot bio‑wastes
Source: Ultrason Sonochem. 2026 Nov 4;135:108145. doi: 10.1016/j.ultsonch.2026.108145 (PMC13769497; doi:10.1016/j.ultsonch.2026.108145)
Supplement: Supplementary file 1 [file mmc1.docx]

**Supplementary Materials**

**Ultrasound‑enhanced green recovery and antioxidant potential of polyphenols from four *Abelmoschus manihot* bio‑wastes**

Wen-zhang Qian^a1^, Li Zhao^a1^, Jin-you Yao^a^, Pei-wen Liang^a^, Yu-han Hu^a^, Yi-xiao Xiao^a^, and Shun Gao^a,b^*

^a^Department of Forestry, Faculty of Forestry, Sichuan Agricultural University, Chengdu, 611130, China

^b^Forest Ecology and Conservation in the Upper Reaches of the Yangtze River Key Laboratory of Sichuan Province & Sichuan Mt. Emei Forest Ecosystem National Observation and Research Station, Sichuan Agricultural University, Chengdu, 611130, China

*Corresponding author: shungao@sicau.edu.cn (Shun Gao)

^1^The authors contributed equally to this work.

**Supplementary Table 1**. Definition of experimental group abbreviations

| Abbreviation | Full description |
| --- | --- |
| NUA-E MS | Non-ultrasound assisted extraction, ethanol monosolvent system |
| UA-E MS | Ultrasonic-assisted extraction, ethanol monosolvent system |
| NUA-W MS | Non-ultrasound assisted extraction, water monosolvent system |
| UA-W MS | Ultrasonic-assisted extraction, water monosolvent system |
| NUA-E/W BS | Non-ultrasound assisted extraction, water/ethanol binary solvent system |
| UA-E/W BS | Ultrasonic-assisted extraction, water/ethanol binary solvent system |

**Supplementary Table 2.** 95% Confidence intervals and RMSE of the kinetic model parameters.

| **Organ** | **System** | **PFO Model** |  |  | **PSO Model** |  |  |
| --- | --- | --- | --- | --- | --- | --- | --- |
|  |  | $k_{1}$95% CI (min⁻¹) | $Y_{e}$95% CI (mg/g) | RMSE | $k_{2}$95% CI (g·mg⁻¹·min⁻¹) | $Y_{e}$95% CI (mg/g) | RMSE |
| **Fruit** | NUA-E MS | [0.177, 0.253] | [4.207, 4.929] | 1.231 | [0.019, 0.079] | [4.693, 5.839] | 0.169 |
|  | UA-E MS | [0.263, 0.328] | [5.111, 5.339] | 0.268 | [0.038, 0.150] | [5.300, 6.094] | 0.153 |
|  | NUA-W MS | [0.082, 0.190] | [12.423, 15.433] | 1.594 | [0.003, 0.013] | [14.892, 19.304] | 0.460 |
|  | UA-W MS | [0.092, 0.219] | [13.518, 15.536] | 1.291 | [0.005, 0.023] | [14.411, 18.545] | 0.573 |
|  | NUA-E/W BS | [0.064, 0.197] | [12.711, 16.425] | 2.210 | [0.002, 0.012] | [15.475, 21.927] | 0.649 |
|  | UA-E/W BS | [0.127, 0.185] | [17.450, 19.251] | 0.773 | [0.007, 0.013] | [20.270, 22.716] | 0.325 |
| **Leaf** | NUA-E MS | [0.146, 0.169] | [9.406, 9.908] | 2.948 | [0.019, 0.032] | [10.266, 11.172] | 0.138 |
|  | UA-E MS | [0.216, 0.266] | [9.681, 10.287] | 0.362 | [0.019, 0.035] | [11.089, 12.131] | 0.168 |
|  | NUA-W MS | [0.066, 0.205] | [12.934, 22.514] | 4.031 | [0.000, 0.005] | [15.227, 43.589] | 1.429 |
|  | UA-W MS | [0.059, 0.141] | [19.421, 26.323] | 1.059 | [0.000, 0.006] | [22.667, 36.981] | 1.095 |
|  | NUA-E/W BS | [0.046, 0.112] | [18.169, 24.465] | 2.334 | [0.000, 0.006] | [19.415, 34.036] | 0.986 |
|  | UA-E/W BS | [0.102, 0.174] | [19.628, 24.291] | 2.636 | [0.002, 0.006] | [25.157, 32.010] | 0.653 |
| **Stem** | NUA-E MS | [0.177, 0.311] | [1.783, 2.307] | 1.036 | [0.016, 0.193] | [2.054, 2.788] | 0.108 |
|  | UA-E MS | [0.205, 0.341] | [2.599, 2.869] | 0.319 | [0.115, 0.185] | [2.972, 3.156] | 0.034 |
|  | NUA-W MS | [0.081, 0.159] | [7.585, 8.475] | 0.917 | [0.006, 0.032] | [7.759, 10.459] | 0.321 |
|  | UA-W MS | [0.067, 0.103] | [10.053, 11.886] | 0.711 | [0.003, 0.008] | [12.553, 16.429] | 0.250 |
|  | NUA-E/W BS | [0.088, 0.438] | [5.734, 7.074] | 0.846 | [0.009, 0.060] | [7.068, 9.106] | 0.312 |
|  | UA-E/W BS | [0.068, 0.142] | [10.371, 14.432] | 1.548 | [0.004, 0.014] | [12.632, 16.795] | 0.414 |
| **Root** | NUA-E MS | [0.039, 0.065] | [1.574, 2.086] | 0.741 | [0.000, 0.095] | [1.326, 2.603] | 0.090 |
|  | UA-E MS | [0.224, 0.448] | [2.009, 2.303] | 0.483 | [0.147, 0.345] | [2.285, 2.497] | 0.042 |
|  | NUA-W MS | [0.058, 0.158] | [8.272, 10.852] | 0.934 | [0.002, 0.018] | [9.377, 14.503] | 0.464 |
|  | UA-W MS | [0.099, 0.151] | [10.039, 10.953] | 1.260 | [0.004, 0.015] | [11.509, 15.027] | 0.335 |
|  | NUA-E/W BS | [0.059, 0.179] | [8.603, 10.907] | 1.432 | [0.003, 0.021] | [9.540, 13.710] | 0.426 |
|  | UA-E/W BS | [0.093, 0.187] | [10.116, 13.048] | 1.699 | [0.005, 0.013] | [13.437, 16.401] | 0.294 |

PFO, pseudo-first-order; PSO, pseudo-second-order; CI, confidence interval; RMSE, root mean square error. 95% CIs were calculated using the standard error derived from the non-linear least-squares regression based on the Student's t-distribution (df = 4, t = 2.776). All calculated lower bounds for that resulted in negative values were constrained to zero for physical meaningfulness.

**Supplementary Table 3.** Coded levels and corresponding actual experimental values of extraction variables.

| Variable | Code Unit | Coded variable level | | |
| --- | --- | --- | --- | --- |
|  |  | -1 | 0 | 1 |
| Ethanol content (%) | A | 15 | 45 | 10 |
| Ultrasound time (min) | B | 5 | 30 | 45 |
| Liquid/solid ratio (mL/g) | C | 25 | 50 | 80 |

**Supplementary Table 4.** BBD and corresponding observed values for TPC, DPPH and ABTS activities of four *A. manihot* bio-wastes under variable UAE parameters.

| Run | Factors | | | Response | | | | | | | | | | | |
| --- | --- | --- | --- | --- | --- | --- | --- | --- | --- | --- | --- | --- | --- | --- | --- |
|  | A: Ethanol content(%) | B: Ultrasound time (min) | C: Liquid/solid ratio (ml/g) | Fruit | | | Leaf | | | Stem | | | Root | | |
|  |  |  |  | TPC  (mg GAE g^-1^) | DPPH (mg Ascorbic acid g⁻¹) | ABTS(mg Ascorbic acid g⁻¹) | TPC  (mg GAE g^-1^) | DPPH (mg Ascorbic acid g⁻¹) | ABTS(mg Ascorbic acid g⁻¹) | TPC  (mg GAE g^-1^) | DPPH (mg Ascorbic acid g⁻¹) | ABTS(mg Ascorbic acid g⁻¹) | TPC  (mg GAE g^-1^) | DPPH (mg Ascorbic acid g⁻¹) | ABTS(mg Ascorbic acid g⁻¹) |
| 1 | -1(15) | 1(55) | 0(45) | 14.54±1.25 | 4.13±0.98 | 5.2±0.81 | 18.67±0.12 | 2.78±0.43 | 5.7±0.03 | 11.13±0.26 | 2.34±0.81 | 4.07±0.65 | 10.83±0.32 | 3.47±0.2 | 3.27±0.17 |
| 2 | 1(75) | -1(5) | 0(45) | 14.78±0.31 | 4.47±0.23 | 3.94±0.09 | 21.61±0.46 | 3.65±0.32 | 5.1±0.05 | 12.89±0.37 | 2.76±0.47 | 3.21±0.07 | 9.8±0.38 | 4.42±0.19 | 3.34±0.09 |
| 3 | 1(75) | 0(30) | 1(80) | 18.08±0.26 | 6.2±0.3 | 5.56±0.22 | 22.95±0.94 | 4.16±0.48 | 6.71±0.1 | 13.62±0.17 | 2.05±0.24 | 4.03±0.15 | 12.95±0.82 | 6.07±0.14 | 4.38±0.17 |
| 4 | 0(45) | 0(30) | 0(45) | 23.37±0.6 | 7.8±0.25 | 6.51±0.07 | 27.37±0.61 | 6.74±0.53 | 7.63±0.12 | 15.83±0.6 | 4.16±0.14 | 4.89±0.11 | 15.5±0.45 | 5.75±0.36 | 5.2±0.14 |
| 5 | 1(75) | 1(55) | 0(45) | 19.6±0.39 | 6.66±0.03 | 5.42±0.19 | 26.11±0.93 | 4.95±0.16 | 6.34±0.14 | 14.54±0.14 | 3.38±0.21 | 4.44±0.12 | 13.28±0.46 | 5.15±0.07 | 4.11±0.15 |
| 6 | 0(45) | 0(30) | 0(45) | 25.19±0.71 | 7.94±0.23 | 6.82±0.29 | 27.55±0.35 | 6.39±0.38 | 7.81±0.1 | 15.53±0.34 | 4.02±0.16 | 5.11±0.27 | 15.31±0.3 | 5.51±0.09 | 5.32±0.13 |
| 7 | 0(45) | 0(30) | 0(45) | 24.55±1.36 | 8.22±0.27 | 6.84±0.14 | 28.13±0.94 | 6.86±0.32 | 8.3±0.18 | 16.15±0.59 | 4.27±0.35 | 5.32±0.23 | 15.46±0.12 | 6.21±0.31 | 5.31±0.24 |
| 8 | 0(45) | 1(55) | 1(80) | 25.22±0.54 | 8.54±0.4 | 8.09±0.44 | 30.22±0.9 | 5.49±0.42 | 8.82±0.3 | 16.38±0.37 | 3.18±0.18 | 5.88±0.24 | 16.95±0.59 | 6.01±0.8 | 5.41±0.31 |
| 9 | 0(45) | 0(30) | 0(45) | 25.4±0.61 | 8.42±1.06 | 7.13±0.21 | 28.45±1.09 | 6.9±0.91 | 8.09±0.18 | 16.43±0.63 | 4.22±0.49 | 5.35±0.31 | 16.02±0.46 | 5.95±0.29 | 5.64±0.13 |
| 10 | 0(45) | 1(55) | -1(10) | 16±0.91 | 6.16±0.25 | 4.53±0.05 | 18.03±1.14 | 5.1±0.33 | 4.49±0.07 | 10.54±0.23 | 3.51±0.17 | 3.77±0.12 | 10.89±0.64 | 3.59±0.31 | 4.14±0.25 |
| 11 | -1(15) | 0(30) | 1(80) | 16.85±0.71 | 5.88±1.18 | 6.72±0.71 | 19.28±0.52 | 2.95±0.79 | 5.92±0.2 | 11.72±0.59 | 2.05±0.12 | 4.43±0.48 | 10.22±0.48 | 4.55±0.23 | 3.47±0.62 |
| 12 | -1(15) | 0(30) | -1(10) | 10.35±0.2 | 3.61±0.12 | 3.98±0.1 | 11.03±0.53 | 3.61±0.15 | 2.8±0.02 | 6.39±0.25 | 2.04±0.02 | 2.91±0.18 | 7.25±0.24 | 1.98±0.02 | 2.87±0.05 |
| 13 | 1(75) | 0(30) | -1(10) | 13.91±0.27 | 5.32±0.14 | 3.79±0.09 | 16.23±1.27 | 3.55±0.17 | 2.93±0.01 | 9.09±0.45 | 2.73±0.17 | 2.89±0.17 | 8.39±0.27 | 2.91±0.08 | 3±0.06 |
| 14 | 0(45) | 0(30) | 0(45) | 24.33±0.72 | 8.47±0.74 | 6.53±0.16 | 29.07±0.55 | 7.03±0.5 | 7.99±0.17 | 16.75±0.9 | 4.36±0.51 | 5.01±0.06 | 15.82±1.02 | 5.75±0.44 | 5.23±0.08 |
| 15 | 0(45) | -1(5) | -1(10) | 14.2±0.11 | 5.5±0.08 | 4.24±0.21 | 17.67±0.11 | 4.77±0.01 | 4.52±0.03 | 9.68±0.42 | 3.1±0.01 | 3.36±0.02 | 9.64±0.36 | 3.16±0.12 | 3.62±0.05 |
| 16 | -1(15) | -1(5) | 0(45) | 12.89±0.62 | 4.46±0.97 | 4.63±0.27 | 18.97±1.04 | 4.27±1.21 | 5.93±0.09 | 10.25±0.64 | 2.41±0.74 | 3.43±0.15 | 9.1±0.51 | 3.98±0.34 | 3.76±0.38 |
| 17 | 0(45) | -1(5) | 1(80) | 19.28±0.82 | 6.88±0.71 | 6.16±0.19 | 24.98±0.9 | 5.75±0.54 | 7.77±0.67 | 14.55±0.73 | 2.7±0.2 | 4.57±0.29 | 13.52±0.62 | 5.65±0.26 | 4.69±0.67 |

Note: Values in parentheses indicate actual experimental values. Data are mean ± SD (three replicates). Five center point runs (Runs 4, 6, 7, 9, 14) are included.

**Supplementary Table 5.** ANOVA and regression statistics for the BBD models describing UAE of polyphenols from fruit of *A. manihot*.

| Source | df | TPC | | | DPPH radical scavenging activity | | | ABTS radical scavenging activity | | |
| --- | --- | --- | --- | --- | --- | --- | --- | --- | --- | --- |
|  |  | Sum of Squares | F-Value | p-Value | Sum of Squares | F-Value | p-Value | Sum of Squares | F-Value | p-Value |
| Model | 9 | 403.73 | 62.90 | < 0.0001*** | 43.16 | 74.08 | < 0.0001*** | 27.15 | 43.75 | < 0.0001*** |
| A | 1 | 17.28 | 24.23 | 0.0017** | 2.63 | 40.58 | 0.0004*** | 0.4160 | 6.03 | 0.0437* |
| B | 1 | 25.23 | 35.38 | 0.0006*** | 2.18 | 33.71 | 0.0007*** | 2.29 | 33.23 | 0.0007*** |
| C | 1 | 77.94 | 109.30 | < 0.0001*** | 5.95 | 91.85 | < 0.0001*** | 12.50 | 181.30 | < 0.0001*** |
| AB | 1 | 2.51 | 3.52 | 0.1027 | 1.59 | 24.51 | 0.0017** | 0.2058 | 2.99 | 0.1276 |
| AC | 1 | 1.36 | 1.91 | 0.2096 | 0.4756 | 7.35 | 0.0302* | 0.2348 | 3.41 | 0.1074 |
| BC | 1 | 4.26 | 5.98 | 0.0444* | 0.2470 | 3.82 | 0.0917 | 0.6705 | 9.73 | 0.0169* |
| A^2^ | 1 | 177.61 | 249.05 | < 0.0001*** | 23.82 | 367.93 | < 0.0001*** | 7.77 | 112.64 | < 0.0001*** |
| B^2^ | 1 | 28.87 | 40.49 | 0.0004*** | 3.12 | 48.12 | 0.0002*** | 1.59 | 23.02 | 0.0020** |
| C^2^ | 1 | 45.14 | 63.30 | < 0.0001*** | 1.22 | 18.80 | 0.0034** | 0.6607 | 9.58 | 0.0174* |
| Residual | 7 | 4.99 |  |  | 0.4532 |  |  | 0.4826 |  |  |
| Lack of Fit | 3 | 2.43 | 1.26 | 0.3993 NS | 0.1075 | 0.4146 | 0.7523 NS | 0.2148 | 1.07 | 0.4557 NS |
| Pure Error | 4 | 2.56 |  |  | 0.3457 |  |  | 0.2677 |  |  |
| C. Total | 16 | 408.72 |  |  | 43.62 |  |  | 27.63 |  |  |
| Std. Dev. |  | 0.8445 | | | 0.2544 | | | 0.2626 | | |
| Mean |  | 18.74 | | | 6.39 | | | 5.65 | | |
| C.V. % |  | 4.51 | | | 3.98 | | | 4.65 | | |
| R^2^ |  | 0.9878 | | | 0.9896 | | | 0.9825 | | |
| Adjusted R² |  | 0.9721 | | | 0.9763 | | | 0.9601 | | |
| Predicted R² |  | 0.8951 | | | 0.9482 | | | 0.8605 | | |
| Adeq Precision |  | 23.1282 | | | 25.2699 | | | 20.8704 | | |
| AICc |  | 84.08 | | | 43.29 | | | 44.36 | | |
| TPC= | 24.57+ | 1.47A+1.78B+3.12C+ | | | 1.03BC-6.49A^2^-2.62B^2^-3.27C^2^ | | |  | | |
| DPPH= | 8.17+ | 0.57A+0.52B+0.86C+ | | | 0.63AB-0.34AC-2.38A^2^-0.86B^2^-0.54C^2^ | | |  | | |
| ABTS= | 6.77- | 0.23A+0.54B+1.25C+ | | | 0.41BC-1.36A^2^-0.61B^2^-0.40C^2^ | | |  | | |

df, degrees of freedom; Std. Dev., standard deviation. C.V. %, coefficient of variation; R², coefficient of determination. Adjusted R², adjusted coefficient of determination; Predicted R², predicted coefficient of determination. Adeq Precision, adequate precision. AICc, corrected Akaike information criterion. Significance levels: * *p* < 0.05, ** *p* < 0.01, *** *p* < 0.001

**Supplementary Table 6.** ANOVA and regression statistics for the BBD models describing UAE of polyphenols from leaf of *A. manihot*.

| Source | df | TPC | | | DPPH radical scavenging activity | | | ABTS radical scavenging activity | | |
| --- | --- | --- | --- | --- | --- | --- | --- | --- | --- | --- |
|  |  | Sum of Squares | F-Value | p-Value | Sum of Squares | F-Value | p-Value | Sum of Squares | F-Value | p-Value |
| Model | 9 | 485.44 | 77.13 | < 0.0001*** | 32.70 | 48.80 | < 0.0001*** | 54.44 | 87.64 | < 0.0001*** |
| A | 1 | 44.91 | 64.21 | < 0.0001*** | 0.9057 | 12.16 | 0.0102* | 0.0674 | 0.9761 | 0.3561 |
| B | 1 | 11.98 | 17.14 | 0.0044** | 0.0023 | 0.0310 | 0.8652 | 0.5167 | 7.49 | 0.0291* |
| C | 1 | 148.64 | 212.54 | < 0.0001*** | 0.2134 | 2.87 | 0.1343 | 26.24 | 380.19 | < 0.0001*** |
| AB | 1 | 5.76 | 8.24 | 0.0240* | 1.95 | 26.20 | 0.0014** | 0.5431 | 7.87 | 0.0263* |
| AC | 1 | 0.5878 | 0.8405 | 0.3898 | 0.3980 | 5.35 | 0.0540 | 0.1095 | 1.59 | 0.2483 |
| BC | 1 | 5.94 | 8.50 | 0.0225* | 0.0853 | 1.15 | 0.3199 | 0.2931 | 4.25 | 0.0783 |
| A^2^ | 1 | 154.89 | 221.47 | < 0.0001*** | 22.06 | 296.24 | < 0.0001*** | 16.87 | 244.35 | < 0.0001*** |
| B^2^ | 1 | 2.14 | 3.06 | 0.1237 | 1.44 | 19.28 | 0.0032** | 0.1583 | 2.29 | 0.1737 |
| C^2^ | 1 | 92.17 | 131.79 | < 0.0001*** | 3.61 | 48.51 | 0.0002*** | 7.90 | 114.48 | < 0.0001*** |
| Residual | 7 | 4.90 |  |  | 0.5212 |  |  | 0.4831 |  |  |
| Lack of Fit | 3 | 2.99 | 2.09 | 0.2445 | 0.2835 | 1.59 | 0.3244 | 0.2170 | 1.09 | 0.4502 |
| Pure Error | 4 | 1.91 |  |  | 0.2377 |  |  | 0.2661 |  |  |
| C. Total | 16 | 490.33 |  |  | 33.22 |  |  | 54.92 |  |  |
| Std. Dev. |  | 0.8363 | | | 0.2729 | | | 0.2627 | | |
| Mean |  | 22.72 | | | 5.00 | | | 6.29 | | |
| C.V. % |  | 3.68 | | | 5.46 | | | 4.18 | | |
| R^2^ |  | 0.9900 | | | 0.9843 | | | 0.9912 | | |
| Adjusted R² |  | 0.9772 | | | 0.9641 | | | 0.9799 | | |
| Predicted R² |  | 0.8964 | | | 0.8523 | | | 0.9292 | | |
| Adeq Precision |  | 29.8855 | | | 18.7519 | | | 29.9176 | | |
| AICc |  | 83.75 | | | 45.67 | | | 44.38 | | |
| TPC= | 28.12+ | 2.37A+1.22B+4.31C+ | | | 1.20AB+1.22BC-6.07A^2^-4.68C^2^ | | |  | | |
| DPPH= | 6.78+ | 0.34A+ | | | 0.70AB-2.29A^2^-0.58B^2^-0.93C^2^ | | |  | | |
| ABTS= | 7.96+ | 0.25B+1.81C+ | | | 0.37AB-2.00A^2^-1.37C^2^ | | |  | | |

df, degrees of freedom; Std. Dev., standard deviation. C.V. %, coefficient of variation; R², coefficient of determination. Adjusted R², adjusted coefficient of determination; Predicted R², predicted coefficient of determination. Adeq Precision, adequate precision. AICc, corrected Akaike information criterion. Significance levels: * *p* < 0.05, ** *p* < 0.01, *** *p* < 0.001

**Supplementary Table 7.** ANOVA and regression statistics for the BBD models describing UAE of polyphenols from Stem of *A. manihot*.

| Source | df | TPC | | | DPPH radical scavenging activity | | | ABTS radical scavenging activity | | |
| --- | --- | --- | --- | --- | --- | --- | --- | --- | --- | --- |
|  |  | Sum of Squares | F-Value | p-Value | Sum of Squares | F-Value | p-Value | Sum of Squares | F-Value | p-Value |
| Model | 9 | 154.41 | 93.23 | < 0.0001*** | 11.11 | 60.47 | < 0.0001*** | 13.18 | 39.88 | < 0.0001*** |
| A | 1 | 14.17 | 77.01 | < 0.0001*** | 0.5393 | 26.42 | 0.0013* | 0.0089 | 0.2433 | 0.6369 |
| B | 1 | 3.41 | 18.53 | 0.0035** | 0.2578 | 12.63 | 0.0093** | 1.61 | 43.75 | 0.0003*** |
| C | 1 | 52.92 | 287.55 | < 0.0001*** | 0.2461 | 12.06 | 0.0104* | 4.47 | 121.73 | < 0.0001*** |
| AB | 1 | 0.1477 | 0.8029 | 0.4000 | 0.1203 | 5.89 | 0.0456* | 0.0875 | 2.38 | 0.1666 |
| AC | 1 | 0.1567 | 0.8514 | 0.3868 | 0.1189 | 5.82 | 0.0465* | 0.0350 | 0.9539 | 0.3613 |
| BC | 1 | 0.2377 | 1.29 | 0.2932 | 0.0018 | 0.0889 | 0.7743 | 0.2026 | 5.52 | 0.0512 |
| A^2^ | 1 | 44.80 | 243.44 | < 0.0001*** | 6.03 | 295.47 | < 0.0001*** | 5.03 | 137.04 | < 0.0001*** |
| B^2^ | 1 | 1.94 | 10.53 | 0.0141* | 0.3520 | 17.25 | 0.0043** | 0.2820 | 7.68 | 0.0276* |
| C^2^ | 1 | 30.17 | 163.96 | < 0.0001*** | 2.67 | 130.77 | < 0.0001*** | 0.9797 | 26.68 | 0.0013** |
| Residual | 7 | 1.29 |  |  | 0.1429 |  |  | 0.2571 |  |  |
| Lack of Fit | 3 | 0.3652 | 0.5276 | 0.6868 | 0.0758 | 1.51 | 0.3412 | 0.0995 | 0.8421 | 0.5377 |
| Pure Error | 4 | 0.9229 |  |  | 0.0671 |  |  | 0.1575 |  |  |
| C. Total | 16 | 155.69 |  |  | 11.25 |  |  | 13.44 |  |  |
| Std. Dev. |  | 0.4290 | | | 0.1429 | | | 0.1916 | | |
| Mean |  | 13.03 | | | 3.13 | | | 4.28 | | |
| C.V. % |  | 3.29 | | | 4.56 | | | 4.48 | | |
| R^2^ |  | 0.9917 | | | 0.9873 | | | 0.9809 | | |
| Adjusted R² |  | 0.9811 | | | 0.9710 | | | 0.9563 | | |
| Predicted R² |  | 0.9532 | | | 0.8829 | | | 0.8632 | | |
| Adeq Precision |  | 30.8580 | | | 20.5853 | | | 20.8387 | | |
| AICc |  | 61.05 | | | 23.67 | | | 33.65 | | |
| TPC= | 16.14+ | 1.33A+0.65B+2.57C- | | | 3.26A^2^-0.68B^2^-2.68C^2^ | | |  | | |
| DPPH= | 4.21+ | 0.26A+0.18B-0.18C+ | | | 0.17AB-0.17AC-1.2A^2^-0.29B^2^-0.8C^2^ | | |  | | |
| ABTS= | 5.14+ | 0.45B+0.75C- | | | 1.09A^2^-0.26B^2^-0.48C^2^ | | |  | | |

df, degrees of freedom; Std. Dev., standard deviation. C.V. %, coefficient of variation; R², coefficient of determination. Adjusted R², adjusted coefficient of determination; Predicted R², predicted coefficient of determination. Adeq Precision, adequate precision. AICc, corrected Akaike information criterion. Significance levels: * *p* < 0.05, ** *p* < 0.01, *** *p* < 0.001

**Supplementary Table 8.** ANOVA and regression statistics for the BBD models describing UAE of polyphenols from root of *A. manihot*.

| Source | df | TPC | | | DPPH radical scavenging activity | | | ABTS radical scavenging activity | | |
| --- | --- | --- | --- | --- | --- | --- | --- | --- | --- | --- |
|  |  | Sum of Squares | F-Value | p-Value | Sum of Squares | F-Value | p-Value | Sum of Squares | F-Value | p-Value |
| Model | 9 | 151.37 | 101.24 | < 0.0001*** | 27.29 | 52.12 | < 0.0001*** | 13.86 | 35.36 | < 0.0001*** |
| A | 1 | 6.17 | 37.13 | 0.0005*** | 2.61 | 44.91 | 0.0003*** | 0.2679 | 6.15 | 0.0422* |
| B | 1 | 12.24 | 73.68 | < 0.0001*** | 0.1265 | 2.17 | 0.1838 | 0.2903 | 6.67 | 0.0364* |
| C | 1 | 38.06 | 229.11 | < 0.0001*** | 14.10 | 242.35 | < 0.0001*** | 2.33 | 53.52 | 0.0002*** |
| AB | 1 | 0.7766 | 4.67 | 0.0674 | 0.3853 | 6.62 | 0.0368* | 0.3927 | 9.02 | 0.0199* |
| AC | 1 | 0.6334 | 3.81 | 0.0918 | 0.0889 | 1.53 | 0.2563 | 0.1503 | 3.45 | 0.1055 |
| BC | 1 | 1.19 | 7.17 | 0.0316* | 0.0013 | 0.0229 | 0.8840 | 0.0101 | 0.2309 | 0.6455 |
| A^2^ | 1 | 65.95 | 396.98 | < 0.0001*** | 5.59 | 96.04 | < 0.0001*** | 8.01 | 183.87 | < 0.0001*** |
| B^2^ | 1 | 3.49 | 20.98 | 0.0025** | 0.7746 | 13.31 | 0.0082** | 0.5014 | 11.52 | 0.0115* |
| C^2^ | 1 | 16.17 | 97.34 | < 0.0001*** | 2.73 | 46.92 | 0.0002*** | 1.20 | 27.47 | 0.0012** |
| Residual | 7 | 1.16 |  |  | 0.4073 |  |  | 0.3048 |  |  |
| Lack of Fit | 3 | 0.8257 | 3.26 | 0.1415 | 0.1376 | 0.6803 | 0.6086 | 0.1816 | 6.15 | 0.0422 |
| Pure Error | 4 | 0.3372 |  |  | 0.2697 |  |  | 0.1232 |  |  |
| C. Total | 16 | 152.53 |  |  | 27.70 |  |  | 14.16 |  |  |
| Std. Dev. |  | 0.4076 | | | 0.2412 | | | 0.2087 | | |
| Mean |  | 12.41 | | | 4.71 | | | 4.28 | | |
| C.V. % |  | 3.28 | | | 5.12 | | | 4.88 | | |
| R^2^ |  | 0.9924 | | | 0.9853 | | | 0.9785 | | |
| Adjusted R² |  | 0.9826 | | | 0.9664 | | | 0.9508 | | |
| Predicted R² |  | 0.9099 | | | 0.9053 | | | 0.7812 | | |
| Adeq Precision |  | 30.9440 | | | 21.1259 | | | 15.3867 | | |
| AICc |  | 59.31 | | | 41.48 | | | 36.55 | | |
| TPC= | 15.62+ | 0.88A+1.24B+2.18C+ | | | 0.55BC-3.96A^2^-0.91B^2^-C^2^ | | |  | | |
| DPPH= | 5.84+ | 0.57A+1.33C+ | | | 0.31AB-1.15A^2^-0.43B^2^-0.81C^2^ | | |  | | |
| ABTS= | 5.34+ | 0.18A+0.19B+0.54C+ | | | 0.31AB-1.38A^2^+0.35B^2^-0.53C^2^ | | |  | | |

df, degrees of freedom. Std. Dev., standard deviation; C.V. %, coefficient of variation. R², coefficient of determination; Adjusted R², adjusted coefficient of determination. Predicted R², predicted coefficient of determination. Adeq Precision, adequate precision; AICc, corrected Akaike information criterion. Significance levels: * *p* < 0.05, ** *p* < 0.01, *** *p* < 0.00

**Supplementary Table 9**. Comparative error analysis of RSM and BP-ANN models.

| Samples | Parameter | MSE | | RMSE | | MAPE (%) | | MAE | |
| --- | --- | --- | --- | --- | --- | --- | --- | --- | --- |
|  |  | RSM | BP-ANN | RSM | BP-ANN | RSM | BP-ANN | RSM | BP-ANN |
| Fruit | TPC | 0.5220 | 0.0125 | 0.7225 | 0.1118 | 3.4782 | 0.2630 | 0.6171 | 0.0458 |
|  | DPPH | 0.0412 | 0.0102 | 0.2030 | 0.1010 | 2.4266 | 0.5496 | 0.1618 | 0.0325 |
|  | ABTS | 0.0543 | 0.0090 | 0.2330 | 0.0949 | 3.5627 | 0.4684 | 0.1965 | 0.0251 |
| Leaf | TPC | 0.5160 | 0.0202 | 0.7183 | 0.1421 | 3.0711 | 0.1085 | 0.6224 | 0.0230 |
|  | DPPH | 0.7222 | 0.0481 | 0.2688 | 0.2193 | 0.2182 | 0.2726 | 4.7865 | 0.0125 |
|  | ABTS | 0.0727 | 0.0056 | 0.2697 | 0.0748 | 3.7311 | 0.6271 | 0.2241 | 0.0352 |
| Stem | TPC | 0.1734 | 0.0026 | 0.4146 | 0.0510 | 2.5840 | 0.0205 | 0.3300 | 0.0025 |
|  | DPPH | 0.0084 | 0.0012 | 0.0916 | 0.0346 | 2.7072 | 0.5301 | 0.0782 | 0.0155 |
|  | ABTS | 0.0347 | 0.0023 | 0.1862 | 0.0480 | 3.9573 | 0.6221 | 0.1676 | 0.0254 |
| Root | TPC | 0.5832 | 0.0015 | 0.7636 | 0.0387 | 5.7101 | 0.7818 | 0.6241 | 0.0910 |
|  | DPPH | 0.0370 | 0.0036 | 0.1922 | 0.0600 | 3.2431 | 1.5443 | 0.1524 | 0.0658 |
|  | ABTS | 0.2530 | 0.0265 | 0.5030 | 0.1628 | 10.2875 | 1.5928 | 0.4071 | 0.0650 |

**Supplementary Table 10.** Annotation confidence, mass accuracy, and repeatability of the 31 representative polyphenols identified in *A. manihot* bio-waste extracts by UHPLC-MS/MS.

| ID | Compounds | Mode | RT (min) | Mass Error (ppm) | MS Score | RSD | Authentic Standard | MSI Level |
| --- | --- | --- | --- | --- | --- | --- | --- | --- |
| 1 | Diosmin | pos | 2.93 | -0.61 | 93 | 0.00129 | Yes | Level 1 |
| 2 | Quercetin | pos | 3.1 | -0.95 | 98.4 | 0.0002 | Yes | Level 1 |
| 3 | Guaiaverin | pos | 3.2 | -0.74 | 96.7 | 0.00031 | Yes | Level 1 |
| 4 | Morin | pos | 3.93 | -0.59 | 91.4 | 0.00045 | Yes | Level 1 |
| 5 | Trans-Ferulic acid | pos | 3.25 | -0.97 | 96.6 | 0.00054 | Yes | Level 1 |
| 6 | Astragalin | pos | 3.23 | -0.92 | 98.9 | 0.00025 | Yes | Level 1 |
| 7 | Cyanidin 3-rutinoside | pos | 3.12 | -1.62 | 98 | 0.00033 | Yes | Level 1 |
| 8 | M-Coumaric acid | pos | 3.07 | -1.08 | 97.7 | 0.00037 | Yes | Level 1 |
| 9 | Rutin | pos | 3 | -0.65 | 96.5 | 0.00029 | Yes | Level 1 |
| 11 | 1,2,3-Trihydroxybenzene | pos | 1.93 | -1.16 | 88.3 | 0.0034 | Yes | Level 1 |
| 12 | Caffeic acid hexoside | neg | 2.46 | -1 | 81 | 0.00031 | Yes | Level 1 |
| 13 | Fertaric acid | neg | 2.62 | -1.81 | 59.5 | 0.00004 | No | Level 2 |
| 14 | Caffeic acid | neg | 2.7 | -1.35 | 97 | 0.0002 | Yes | Level 1 |
| 15 | Ellagic acid | neg | 3.04 | -0.8 | 82 | 0.00086 | Yes | Level 1 |
| 16 | 4-Hydroxycinnamic acid | neg | 3.07 | -1.55 | 99.1 | 0.00024 | Yes | Level 1 |
| 17 | Isoquercitrin | neg | 3.1 | -1.3 | 96.8 | 0.00038 | Yes | Level 1 |
| 18 | Kaempferol | neg | 4.31 | -1.32 | 89.5 | 0.00015 | Yes | Level 1 |
| 19 | Lappaol A | neg | 5.14 | -1.1 | 86.5 | 0.0002 | Yes | Level 1 |
| 20 | Kadangustin D | neg | 6.78 | 1.51 | 89.4 | 0.00199 | Yes | Level 1 |
| 21 | Kadsuphilol B | neg | 4.62 | -1.21 | 85.9 | 0.00027 | Yes | Level 1 |
| 22 | Tricetin | neg | 3.93 | -1.1 | 89.7 | 0.00029 | Yes | Level 1 |
| 23 | Buddlenoid A | neg | 3.83 | -0.94 | 81.1 | 0.00014 | No | Level 2 |
| 24 | Naringin dihydrochalcone | neg | 3.55 | -0.03 | 85.5 | 0.00109 | No | Level 2 |
| 25 | 3-O-Methylellagic acid | neg | 3.52 | -1.24 | 80.9 | 0.00167 | Yes | Level 1 |
| 26 | Cis-Coutaric acid | neg | 2.32 | -1.97 | 88.6 | 0.00076 | No | Level 2 |
| 27 | Tichocarpol A | neg | 2.09 | -1.11 | 66.6 | 0.0002 | Yes | Level 1 |
| 28 | 3,4-Dihydroxybenzoic acid | neg | 2.08 | -1.17 | 93.1 | 0.00042 | Yes | Level 1 |
| 29 | Beta-Glucogallin | neg | 1.91 | 0.1 | 91.9 | 0.00118 | Yes | Level 1 |
| 30 | Gallic acid | neg | 1.13 | -1.34 | 99.5 | 0.00042 | Yes | Level 1 |
| 31 | Hyperin | neg | 3.1 | -1.44 | 95.8 | 0.00142 | Yes | Level 1 |

MSI Level 1 indicates unambiguous identification confirmed by authentic standards (matching both retention time and MS² fragments); Level 2 indicates confident identification based on accurate mass (< 2 ppm) and high-quality database matching without standard verification. MS Score represents the MS² fragmentation matching score against the reference databases. RSD (Relative Standard Deviation) was calculated from Quality Control (QC) samples to evaluate instrument precision; all values were well below the 30% threshold, demonstrating high data stability.

**Supplementary Fig. 1**. Resource recycling of different bio-wastes of *A. manihot*. (A) The commercially applied flower part and discarded bio-waste including leaf, stem, root and fruit of *A. manihot*. (B) The proportion of different bio-wastes of *A. manihot*, where waste biomass accounts for over 80% of the whole plant. (C) Chemical structures of four typical dominant polyphenolic monomers abundant in *A. manihot* bio-wastes, namely quercetin, kaempferol, caffeic acid and gallic acid. (D) Schematic workflow of the full research programme from solvent screening to the final establishment of polyphenol recycling system.

**Supplementary Fig. 2.** RSM plots for the optimization of UAE parameters from fruit bio-wastes of *A. manihot*. (A–C) Response surfaces for DPPH radical scavenging activity. (D–F) Response surfaces for ABTS radical scavenging activity. Plots illustrate the combined effects of (A, D) ethanol content and ultrasound time, (B, E) ethanol content and LSR, and (C, F) ultrasound time and LSR

**.**

**Supplementary Fig. 3** RSM plots for the optimization of UAE parameters from leaf bio-wastes of *A. manihot*. (A–C) Response surfaces for DPPH radical scavenging activity. (D–F) Response surfaces for ABTS radical scavenging activity. Plots illustrate the combined effects of (A, D) ethanol content and ultrasound time, (B, E) ethanol content and LSR, and (C, F) ultrasound time and LSR

**Supplementary Fig. 4.** RSM plots for the optimization of UAE parameters from stem bio-wastes of *A. manihot*. (A–C) Response surfaces for DPPH radical scavenging activity. (D–F) Response surfaces for ABTS radical scavenging activity. Plots illustrate the combined effects of (A, D) ethanol content and ultrasound time, (B, E) ethanol content and LSR, and (C, F) ultrasound time and LSR.

**Supplementary Fig. 5.** RSM plots for the optimization of UAE parameters from root bio-wastes of *A. manihot*. (A–C) Response surfaces for DPPH radical scavenging activity. (D–F) Response surfaces for ABTS radical scavenging activity. Plots illustrate the combined effects of (A, D) ethanol content and ultrasound time, (B, E) ethanol content and LSR, and (C, F) ultrasound time and LSR.

**Supplementary Fig. 6.** BBD cube plots showing the effects of ethanol content, ultrasound time, and LSR on TPC (A–D), DPPH radical scavenging activity (E–H), and ABTS radical scavenging activity (I–L) of extracts from different bio-wastes of *A. manihot*. (A, E, I) Fruit, (B, F, J) Leaf, (C, G, K) Stem, (D, H, L) Root.

**Supplementary Fig. 7.** Contour plots corresponding to the RSM optimization of UAE parameters for fruit samples of *A. manihot.* (A–C) Contour plots for TPC. (D–F) Contour plots for DPPH radical scavenging activity. (G–I) Contour plots for ABTS radical scavenging activity. The plots depict the combined effects of (A, D, G) ethanol content and ultrasound time, (B, E, H) ethanol content and LSR, and (C, F, I) ultrasound time and LSR.

**Supplementary Fig. 8.** Contour plots corresponding to the RSM optimization of UAE parameters for leaf samples of *A. manihot.* (A–C) Contour plots for TPC. (D–F) Contour plots for DPPH radical scavenging activity. (G–I) Contour plots for ABTS radical scavenging activity. The plots depict the combined effects of (A, D, G) ethanol content and ultrasound time, (B, E, H) ethanol content and LSR, and (C, F, I) ultrasound time and LSR.

**Supplementary Fig. 9.** Contour plots corresponding to the RSM optimization of UAE parameters for stem samples of *A. manihot.* (A–C) Contour plots for TPC. (D–F) Contour plots for DPPH radical scavenging activity. (G–I) Contour plots for ABTS radical scavenging activity. The plots depict the combined effects of (A, D, G) ethanol content and ultrasound time, (B, E, H) ethanol content and LSR, and (C, F, I) ultrasound time and LSR.

**Supplementary Fig. 10.** Contour plots corresponding to the RSM optimization of UAE parameters for root samples of *A. manihot.* (A–C) Contour plots for TPC. (D–F) Contour plots for DPPH radical scavenging activity. (G–I) Contour plots for ABTS radical scavenging activity. The plots depict the combined effects of (A, D, G) ethanol content and ultrasound time, (B, E, H) ethanol content and LSR, and (C, F, I) ultrasound time and LSR.

**Supplementary Fig. 11.** Box-Cox power transformation plots for model residuals of the RSM models developed for UAE parameters optimization from different parts of *A. manihot*. (A–D) Plots for TPC of fruit, leaf, stem, and root, respectively. (E–H) Plots for DPPH radical scavenging activity of fruit, leaf, stem, and root, respectively. (I–L) Plots for ABTS radical scavenging activity of fruit, leaf, stem, and root, respectively. The green vertical line indicates the optimal λ value minimizing the residual sum of squares, while the horizontal line and the red/blue dashed lines denote the 95% confidence interval.

**Supplementary Fig. 12.** Predicted *vs.* actual values plots for the RSM models. (A–D) Correlations between predicted and experimental values for TPC from fruit (A), leaf (B), stem (C), and root (D) samples. (E–H) Correlations for DPPH radical scavenging activity from fruit (E), leaf (F), stem (G), and root (H) samples. (I–L) Correlations for ABTS radical scavenging activity from fruit (I), leaf (J), stem (K), and root (L) samples.

**Supplementary Fig. 13.** ANN modeling and training for the UAE of polyphenols from *A. manihot* fruit. (A) Schematic architecture of the ANN model. (B) Regression analysis plots showing the correlation between predicted and target values for training, validation, test, and all datasets. (C) MSE performance of the ANN model during training, validation, and testing epochs. (D) Error histogram illustrating the distribution of prediction errors (targets - outputs) across all datasets.

**Supplementary Fig. 14.** ANN modeling and training for the UAE of polyphenols from *A. manihot* leaf. (A) Schematic architecture of the ANN model. (B) Regression analysis plots showing the correlation between predicted and target values for training, validation, test, and all datasets. (C) MSE performance of the ANN model during training, validation, and testing epochs. (D) Error histogram illustrating the distribution of prediction errors (targets - outputs) across all datasets.

**Supplementary Fig. 15.** ANN modeling and training for the UAE of polyphenols from *A. manihot* stem. (A) Schematic architecture of the ANN model. (B) Regression analysis plots showing the correlation between predicted and target values for training, validation, test, and all datasets. (C) MSE performance of the ANN model during training, validation, and testing epochs. (D) Error histogram illustrating the distribution of prediction errors (targets - outputs) across all datasets.

**Supplementary Fig. 16.** ANN modeling and training for the UAE of polyphenols from *A. manihot* root. (A) Schematic architecture of the ANN model. (B) Regression analysis plots showing the correlation between predicted and target values for training, validation, test, and all datasets. (C) MSE performance of the ANN model during training, validation, and testing epochs. (D) Error histogram illustrating the distribution of prediction errors (targets - outputs) across all datasets.

**Supplementary Fig. 17.** Experimental MS/MS fragmentation spectra of the 31 representative polyphenolic compounds identified in A. manihot bio-waste extracts. The x-axis indicates the mass-to-charge ratio (m/z), and the y-axis indicates the relative intensity (%).

**Supplementary Fig. 18.** MS/MS mirror plots of the 26 representative polyphenolic compounds identified in A. manihot bio-waste extracts. For each compound, the upper panel (experiment) shows the experimental MS/MS fragmentation spectrum, and the lower panel (database) displays the matched reference spectrum from the corresponding database (e.g., NIST 2023, MJDB). Red vertical bars represent matched fragment peaks, while grey bars indicate unmatched peaks. The experimental precursor m/z (EXP) and the matched database ID (DB) are provided in the top-left legend of each panel.

**Supplementary Fig. 19.** Hierarchical clustering heatmap of top abundant polyphenolic metabolites annotated from four different tissues (Root, Leaf, Stem, Fruit) of *A. manihot* *via* UHPLC-MS/MS metabolomics. Color gradient from blue to red represents the normalized relative abundance ranging from low to high.

**Supplementary Fig. 20.** (A) Hierarchical clustering analysis of the top 50 most abundant flavonoids. (B Hierarchical clustering analysis of the top 50 most abundant phenolic acids. (C) Hierarchical clustering analysis of tannins identified in the extracts.

**Supplementary Fig. 21.** Volcano plots of differentially abundant polyphenolic metabolites among different parts of *A. manihot*. The volcano plots display the relationship between the log₂ fold change (log₂FC, x-axis) and the negative logarithm of the *p*-value (-log₁₀(p-value), y-axis). Significantly upregulated metabolites (log₂FC > 2, p ≤ 0.05) are highlighted in red ("Up"), significantly downregulated metabolites (log₂FC < -2, p ≤ 0.05) in blue ("Down"), and non-significant metabolites in grey ("NoDiff"). (A) Fruit *vs*. Leaf; (B) Fruit *vs*. Stem; (C) Root *vs*. Fruit; (D) Stem *vs*. Leaf; (E) Root *vs*. Stem; (F) Leaf *vs*. Root.
